# Human stem cell-based embryo models: innovation, ethics, and policy

**DOI:** 10.1093/humrep/deag035

**Published:** 2026-03-24

**Authors:** Alfonso Martinez Arias, Nicolas Rivron, Shahragim Tajbakhsh, Josephine Johnston, Cantas Alev, Laure Bally-Cuif, Elvan Böke, Tommaso Cavazza, Emma Cave, David Cyranoski, Laurent David, Miguel A Esteban, Jianping Fu, Niels Geijsen, Nienke de Graeff, Jacob H Hanna, Nick Hopwood, Maneesha S Inamdar, Fredrik Lanner, Brigitte Leeners, Zhen Liu, Maitre Jean Leon, Gabriella Minchiotti, Naomi Moris, Megan Munsie, Kathy K Niakan, Olivier Pourquie, Vincent Pasque, Martin Pera, Yaojin Peng, Sophie Petropoulos, Sharad Ramanathan, Janet Rossant, Peter Rugg-Gunn, Mitinori Saitou, Karen Sermon, Jose Silva, Thorold Theunissen, Margherita Turco, John Wallingford, Hongmei Wang, Aryeh Warmflash, Jun Wu, Leqian Yu

**Affiliations:** Institució Catalana de Recerca i Estudis Avançats (ICREA) and MELIS Universitat Pompeu Fabra, Barcelona, Spain; Institute of Molecular Biotechnology of the Austrian Academy of Sciences (IMBA), Vienna BioCenter (VBC), Vienna, Austria; Department of Developmental and Stem Cell Biology, Stem Cells and Development Unit, Institut Pasteur, Université Paris Cité, CNRS UMW 3738, Paris, France; Department of Bioethics, Faculty of Medicine, University of Otago, Dunedin, New Zealand; Institute for the Advanced Study of Human Biology (ASHBi), Kyoto University, Kyoto, Japan; Institut Pasteur, Université Paris Cité, CNRS UMR3738, Zebrafish Neurogenetics Unit, Paris, France; Institució Catalana de Recerca i Estudis Avançats (ICREA) and CRG BIST, Barcelona, Spain; Department of Reproductive Endocrinology, University Hospital Zurich, University of Zurich, Zurich, Switzerland; Durham Law School, Durham University, Durham, UK; Institute for the Advanced Study of Human Biology (ASHBi), Kyoto University, Kyoto, Japan; Nantes University, CHU Nantes, Inserm, CR2TI, Nantes, France; CHU Nantes, Service de Biologie de la Reproduction, Nantes, France; 3DC STAR Lab, BGI CELL, Shenzhen, China; Prince Fahad bin Sultan Research Chair for Biomedical Research, University of Tabuk, Tabuk, Saudi Arabia; Department of Biomedical Engineering, University of Michigan, Ann Arbor, MI, USA; Department of Anatomy and Embryology, Leiden University Medical Center, Leiden, The Netherlands; The Novo Nordisk Foundation Center for Stem Cell Medicine (reNEW), Leiden Node, Leiden, The Netherlands; The Novo Nordisk Foundation Center for Stem Cell Medicine (reNEW), Leiden Node, Leiden, The Netherlands; Department of Medical Ethics & Health Law, Leiden University Medical Center, Leiden, The Netherlands; Department of Molecular Genetics, Weizmann Institute of Science, Rehovot, Israel; Department of History and Philosophy of Science, University of Cambridge, Cambridge, UK; Centre for Research Application and Training in Embryology (CReATE), Institute for Stem Cell Science and Regenerative Medicine (BRIC-inStem), Bangalore, India; Jawaharlal Nehru Centre for Advanced Scientific Research (JNCASR), Bangalore, India; Department of Gynecology and Reproductive Medicine, Karolinska University Hospital, Stockholm, Sweden; Division of Obstetrics and Gynecology, Department of Clinical Science, Intervention and Technology, Karolinska Institutet, Karolinska Universitetssjukhuset, Stockholm, Sweden; Department of Reproductive Endocrinology, University Hospital Zurich, University of Zurich, Zurich, Switzerland; Primate lab Institute of Neuroscience, CAS, Shanghai, China; Institut Curie, Université PSL, CNRS UMR3215, INSERM U934, Paris, France; Stem Cell Fate Laboratory, Institute of Genetics and Biophysics “A. Buzzati Traverso”, National Research Council, Naples, Italy; The Francis Crick Institute, London, UK; Stem Cell Ethics & Policy Group, Murdoch Children’s Research Institute, Parkville, VIC, Australia; Department of Paediatrics, University of Melbourne, Parkville, VIC, Australia; Loke Centre for Trophoblast Research, Department of Physiology, Development and Neuroscience, University of Cambridge, Cambridge, UK; Department of Genetics, Harvard Medical School, Boston, MA, USA; Department of Pathology, Brigham and Women’s Hospital, Boston, MA, USA; Department of Development and Regeneration, Leuven Institute for Single Cell Omics (LISCO), KU Leuven, Leuven, Belgium; The Jackson Laboratory, Mammalian Genetics, Bar Harbor, ME, USA; State Key Laboratory of Stem Cell and Reproductive Biology, Institute of Zoology, Chinese Academy of Sciences, Beijing, China; Institute for Stem Cell and Regeneration, Chinese Academy of Sciences, Beijing, China; Department of Gynecology and Reproductive Medicine, Karolinska University Hospital, Stockholm, Sweden; Division of Obstetrics and Gynecology, Department of Clinical Science, Intervention and Technology, Karolinska Institutet, Karolinska Universitetssjukhuset, Stockholm, Sweden; Centre de Recherche du Centre Hospitalier, Université de Montréal, Montréal, Canada; Département de Médecine, Molecular Biology Programme, Université de Montréal, Montréal, Canada; Departments of Molecular and Cellular Biology, Stem Cell and Regenerative Biology, Applied Physics, Harvard University, Cambridge, MA,USA; Department of Neurology, Massachusetts General Hospital, Charlestown, MA, USA; The Gairdner Foundation and the Hospital for Sick Children, University of Toronto, Toronto, Canada; Babraham Institute (UK) Epigenetics Programme, Babraham Institute, Cambridge, UK; Cambridge Stem Cell Institute, University of Cambridge, Cambridge, UK; Institute for the Advanced Study of Human Biology (ASHBi), Kyoto University, Kyoto, Japan; Department of Anatomy and Cell Biology, Graduate School of Medicine, Kyoto University, Sakyo-ku, Kyoto, Japan; Center for iPS Cell Research and Application (CiRA), Kyoto University, Sakyo-ku, Kyoto, Japan; Research Group Genetics Reproduction and Development (GRAD), Vrije Universiteit Brussel, Brussels, Belgium; Guangzhou National Laboratory, Guangzhou International Bio Island, Guangzhou, Guangdong, China; Department of Developmental Biology and Center of Regenerative Medicine, Washington University School of Medicine, St. Louis, MO, USA; Friedrich Miescher Institute for Biomedical Research, Basel, Switzerland; Department of Molecular Biosciences, University of Texas at Austin, Austin, TX, USA; Key Laboratory of Organ Regeneration and Reconstruction, State Key Laboratory of Stem Cell and Reproductive Biology, Institute of Zoology, Chinese Academy of Sciences, Beijing, China; University of Chinese Academy of Sciences, Beijing, China; Beijing Institute for Stem Cell and Regenerative Medicine, Beijing, China; Division of Developmental Biology, Center for Stem Cell & Organoid Medicine (CuSTOM), Cincinnati Children’s Hospital, Cincinnati, OH, USA; Department of Molecular Biology, University of Texas Southwestern Medical Center, Dallas, TX, USA; Hamon Center for Regenerative Science and Medicine, University of Texas Southwestern Medical Center, Dallas, TX, USA; Ida Green Center for Reproductive Biology Sciences, University of Texas Southwestern Medical Center, Dallas, TX, USA; Key Laboratory of Organ Regeneration and Reconstruction, State Key Laboratory of Stem Cell and Reproductive Biology, Institute of Zoology, Chinese Academy of Sciences, Beijing, China; University of Chinese Academy of Sciences, Beijing, China; Beijing Institute for Stem Cell and Regenerative Medicine, Beijing, China

**Keywords:** human embryos, SCBEMs, *in vitro* fertilization, implantation, blastocyst, gastrulation, ethics, pluripotent stem cells, embryogenesis, gametes

## Abstract

The aim of this White Paper is to establish a foundational framework for *research*, *technological development*, *and regulation* in the emerging field of stem cell-based embryo models (SCBEMs). These models, generated from Pluripotent Stem Cells, are designed to recapitulate essential events in early stages of human development. They have the potential to illuminate the early stages of embryo development and implantation and hold promise as an avenue to address global health challenges, including infertility and pregnancy loss, congenital, neonatal and adult conditions, and the need for organ transplants. While SCBEMs are not a substitute for human embryos, their tractability for large-scale analysis and their abilities to model the earliest stages of embryonic development suggest that they will have a significant impact on reproductive biology and regenerative medicine. But SCBEMs do not just raise novel scientific questions; they pose ethical and legal questions that need to be addressed. The paper stems from a meeting of a core group of researchers that met at the Institut Pasteur in Paris in November 2024 and represents the views of an extended group that has worked to elaborate the documents as a consensus for the field. Here, we provide a framework to guide research in this new field. We do this by summarizing the state of the science, assessing current SCBEM research in relation to its primary future applications and addressing the need for continued ethical and regulatory oversight associated with this new field.

## The need for research on embryonic development

The earliest stages of human development, from the first cell divisions to implantation (see Table 1) in the uterine lining and the establishment of the body plan (see Table 1) through gastrulation (see Table 1), are crucial for a successful pregnancy and the long-term health of the newborn. This is also a fragile period. Many people struggle to become pregnant, with infertility affecting around 15% of couples worldwide, an estimated total of 48 million couples (The Lancet Global Health, 2022). Although success rates for ART are improving, they are only around 30–35% (Wade *et al*., 2015; Human Fertilisation and Embryology Authority UK, 2025). Furthermore, only about a third of fertilized eggs progress beyond the third week of development (Wilcox *et al*., 2001; Larsen *et al*., 2013; Li *et al*., 2015), and ∼3% of newborn babies have congenital conditions, which prove lethal for 17/1000 infants (Li *et al*., 2025); conditions include cardiovascular and metabolic disorders, limb abnormalities, and sensory deprivation. In addition, several serious complications in pregnancy, such as pre-eclampsia (Jung *et al*., 2022) and foetal growth restriction (Burton *et al*., 2016), are thought to have their origins in events that occur during the first 3 weeks of embryonic development (Smith, 2010). The incidence of these disorders is rising globally (Bai *et al*., 2024). These health challenges and others underscore the pressing need to advance understanding of human reproductive biology and embryology.

**Table 1.** **Definitions of terms used in the text**.

**Blastoid**, a stem cell-derived structure that resembles a blastocyst in morphology and gene expression but is generated *in vitro* from PSCs rather than through fertilization, parthenogenesis, or somatic cell nuclear transfer.
**Blastocyst**, a pre-implantation-stage embryo formed around 5–6 days after fertilization in humans. It is characterized by a fluid-filled cavity (blastocoel), an outer layer of trophectoderm cells (which contribute to the placenta), and an inner cell mass comprised of epiblast and hypoblast cells (which give rise to the embryo proper and to extraembryonic membranes of the conceptus, respectively). In humans, a blastocyst represents the last early embryonic developmental stage that is still competent to implant in utero upon embryo transfer.
**Body plan** refers to the basic layout or blueprint of an organism’s structure, established during embryonic development (largely through gastrulation and subsequent events), including axes (anterior–posterior, dorsal–ventral) that serve as a reference to organize the primordia of the different tissues and organs.
**Conceptus** refers to the ensemble of the embryonic and extraembryonic cells and tissues.
**Embryo** refers to the early developmental stages of a multicellular organism following the fertilization of an egg or an equivalent process such as Somatic Cell Nuclear Transfer (SCNT).
**Embryonic stem cells (ESC)** are a type of stem cell originally derived from the inner cell mass of a mammalian blastocyst that can proliferate indefinitely in culture and retain the ability to specialize into all cell lineages of the body. There are two types of ESCs: naive and primed ESCs. Naive ESCs can give rise to the three lineages that configure the blastocyst, whereas primed ESCs are pluripotent. Under special culture conditions, human embryonic stem cells (ESC) can also produce extraembryonic cell types including trophoblast cells.
**Endometrium**, a membrane that lines up the uterus that thickens during the menstrual cycle and provides the cellular elements for the implantation of the blastocyst.
**Epiblast**, the layer of cells in an embryo that results from further development of the inner cell mass of the blastocyst during implantation. During gastrulation, the epiblast gives rise to the amniotic membrane as well as to the three germ layers (ectoderm, mesoderm and endoderm) that represent the groups of cells of origin for the different tissues and organs and the amnion.
**Extraembryonic layers** are multicellular elements that do not contribute to the embryo. They include two lineages that are segregated prior to implantation and that are integrated in the blastocyst: the trophectoderm that will form the bulk of the placenta, and the Primitive Endoderm, that will form the yolk sac and play a role in the early patterning of the epiblast. A third extraembryonic tissue is the amnion that having an origin in the embryonic tissue, does not contribute to the embryo but forms a protective wrapping to maintain the embryo in a liquid environment.
**Gastrulation**, a fundamental process in early embryonic development whereby the epiblast acquires a coordinated system of spatial organization as it develops the three primary germ layers: ectoderm, mesoderm, and endoderm. In humans, this process occurs approximately between Days 14 and 21 post-fertilization, marking a key transition in the elaboration of the body plan. It is characterized by the presence of a primitive streak.
**Gastruloid**, an *in vitro* structure derived from PSCs that simulates certain aspects of gastrulation (e.g. symmetry breaking and germ-layer patterning) and contains derivatives of more than one germ layer. It lacks extraembryonic tissues and does not give rise to a complete embryo, Now, gastruloids cover a developmental period from the beginning of gastrulation, Day 14, to the start of somitogenesis, Day 21.
**Implantation,** in humans, it refers the process whereby the blastocyst attaches and invades the endometrium to establish a connection with maternal tissues that will sustain the development of the embryo and the foetus.
***In vitro* gametogenesis** is the generation of functional gametes in vitro from PSCs.
**Induced pluripotent stem cells** are a type of PSC generated by reprogramming adult somatic cells (e.g. skin fibroblasts) to a pluripotent state, often through the expression of defined transcription factors, or through defined exogenously added molecules.
**Pluripotent stem cells** are any stem cell (embryonic or induced) with the capacity to differentiate into all cell types of the adult organism.
**Primitive streak**, a furrow that appears at the posterior midline of the epiblast and marks the start of gastrulation. It is a dynamic structure that outlines the anterior–posterior axis of the embryo and is the seed of all cell types except for the brain. Its formation is a landmark associated with the ‘14 day rule’ which, in many countries, signals the current limit for the culture of human embryos *in vitro*.
**Stem cell-based embryo models** are an organized, 3-dimensional structure derived wholly or primarily from PSCs that aims to replicate features of embryonic development. Depending on the conditions and cell types used to create SCBEM, they can adopt a wide range of forms that aim to model different stages of embryonic development. Common examples of SCBEMs include blastoids and gastruloids.
**Totipotent stem cells** are an embryonic stem cell with the capacity to give rise to an organism. However, recently the term is being used to refer to a cell gives rise to the three lineages of a blastocyst.
**Zygote** is the single cell formed immediately upon fertilization of the egg by the sperm. A zygote has the potential to give rise to an embryo.

The development of IVF in the 1970s included methods to extract, mature, and fertilize oocytes and, crucially, to culture embryos (see Table 1) *ex vivo* until the blastocyst (see Table 1) stage (Steptoe *et al*., 1971).

IVF opened up the opportunity to study the earliest stages of human development in the laboratory (Hopwood, 2024), including gastrulation, the stage in development when the epiblast (see Table 1), an epithelium that results from the cleavage and growth of the embryonic component of the blastocyst, generates the outline of the organism (Stern, 2004). In humans, the process commences around Day 14 after fertilization and is completed a week later (Ghimire *et al*., 2021; O’Rahilly *et al*., 1981). Gastrulation is a complex process tightly associated with the emergence of the primitive streak (see Table 1), a groove that outlines the main body axis of the organism. It involves a suite of organized cell movements and the orderly emergence of the primordia for the different tissues and organs along the anterior to posterior axis.

Although embryo attrition happens principally before or at implantation, many congenital anomalies have their origins during gastrulation (Moore *et al*., 2020; Abas *et al*., 2022) as this process involves a complex interplay between elements of the three germ layers, shaping the emergence of tissues and organs. The cardiac system, for example, requires a large number of spatial and temporal interactions between different cell populations and thus is particularly prone to undergo errors that will lead to structural abnormalities (Wu *et al*., 2020; Xu *et al*., 2025); between 0.8 and 1% of newborns exhibit congenital cardiovascular disorders. Gastrulation is also a period during which the developing embryo is most sensitive to teratogens and toxins and sensitive to lifestyle factors (Sadler, 2017). Presently, research on the impact of these substances relies on a variety of mainly non-human animal systems (Alves-Pimenta *et al*., 2024). Therefore, experimental models of human embryonic development are needed to reflect more accurately human-specific biological processes and responses.

Currently, studies on the early stages of human development, along with research focused on improving the success of IVF and related ART, rely on access to donated human embryos from fertility clinics. The supply of such embryos is limited and subject to ethical and legal constraints. In several countries, research on human embryos is prohibited altogether, while in many others, research is limited by the 14-day rule (Warnock, 1984; Franklin, 2019). First proposed in 1979 by the United States Department of Health, Education and Welfare in response to the development of IVF, it was articulated in its most familiar form in 1984 by the Warnock committee in the UK (Wallace, 2017). The 14-day rule prohibits the culture of a human embryo beyond Day 14 of development or the appearance of the primitive streak, a sign of gastrulation. These markers are often taken to indicate the beginning of an individual to a degree that merits legal protection. The rule has been widely adopted, including in law in many countries, for example, in the UK’s Human Fertilisation and Embryology Act 1990 (Franklin, 2019).

Recently, new culture systems have enabled *in vitro* development of human blastocysts to Day 14 (Deglincerti *et al*., 2016; Shahbazi *et al*., 2016) and of non-human primates (NHPs) beyond the gastrulation stage (Gong *et al*., 2023; Zhai *et al*., 2023). These advances have led some researchers, advocacy groups, ethicists, and lawyers to argue for extending the 14-day rule to enable research on the establishment of the body plan and early organogenesis (Hyun *et al*., 2016; Pera, 2017). As a result of these calls, several jurisdictions are considering changes to their law or policy in this area. A committee appointed by the Dutch Health Council, for example, has recommended allowing supervised research on human embryos beyond 14 days on the basis of ‘the valuable information to be obtained from embryo research’ (M’Hamdi and de Wert, 2024) and the Nuffield Council of Bioethics in the UK is also considering an extension (Nuffield Council on Bioethics, 2017). While it is not yet clear whether this recommendation will be implemented by the Dutch government, support for changing the 14-day rule is rising internationally.

## Pluripotent stem cell-based models and their advantages for studies of early human development

Notwithstanding developments in the culture of embryos beyond the blastocyst stage in the lab and the potential extension of the 14-day rule, continued reliance on surplus ART embryos for studies of early development has inherent shortcomings, and there is close to no access to embryos at implantation and peri-gastrulation stages (Rugg-Gunn *et al*., 2023). Furthermore, given the high rate of attrition during the first 2 weeks of development, it is difficult to assess whether observed abnormalities of post-implantation development *in vitro* are intrinsic to the embryo, the quality of the zygote (see Table 1), or caused by the experimental procedure. Our limited knowledge of the gene and cell regulatory networks underlying the early stages of human development, blastocyst formation, and implantation adds to the uncertainties raised by these studies.

Although NHPs provide useful alternative models that can be used as a reference for human development, there are key differences between humans and these species in how embryos implant and the timing of embryo development (Siriwardena and Boroviak, 2022). This means that significant developmental events need to be studied in human embryos, where research is legally restricted. Taking these limitations into consideration, recent developments in the uses of Pluripotent Stem Cells (PSCs) offer an alternative for the study of the early stages of human development.

Derived either from pre-implantation embryos (Embryonic Stem Cells, ESCs; see Table 1) or from adult cells reprogrammed into a pluripotent state (induced Pluripotent Stem Cells, iPSCs; see Table 1), PSCs can self-renew indefinitely *in vitro* while retaining the ability to differentiate into all cell types of the organism (Wang and Wu, 2022; Smith, 2024). In adherent culture, both PSCs and iPSCs can be steered to form specific cell types that, in some instances, can be used in clinical applications. Over the last few years, several cell types have been developed in this manner that are undergoing clinical trials in regenerative medicine (Kirkeby *et al*., 2025).

The ability to differentiate PSCs *in vitro* into different cell types paved the way for studies leading to the establishment of models of early embryonic development (Shahbazi *et al*., 2019; Fu *et al*., 2021) dubbed stem cell-based embryo models (SCBEMs; see Table 1) (Martinez Arias *et al*., 2024). These models represent various stages of embryonic development, ranging from pre-implantation to early post-implantation periods, including peri-gastrulation stages.

Human SCBEMs have significant differences from embryos but also several research advantages over human embryos. It is possible to generate them in large numbers, allowing screens that are not possible with the current supply of IVF embryos. In addition, human SCBEMs created using iPSCs derived from the cells of patients with specific diseases or genetic conditions open opportunities for disease modelling and, in the long term, regenerative medicine.

## The need for a SCBEM framework

In response to the development of SCBEMs and the questions they raise, we convened a working group that met in person at the Institut Pasteur, Paris (France), in November 2024 and later several times online, including people who could not attend the in-person meeting. This paper summarizes the discussions of the extended working group and provides a framework to guide research in this new field. It is organized into three key sections:

- **SCBEMs state of the science and the need for standards—**outlining the urgent biological and clinical questions that SCBEMs may help to answer and establishing the imperative for standardization.
- **Emergent technologies and applications—**highlighting the latest methods used to create and use SCBEMs.
- **Ethical and legal considerations in SCBEM research—**detailing the evolving ethical debate around SCBEMs and the regulatory frameworks needed to guide their use, and proposing strategies to enhance transparency, public understanding, and trust in SCBEM research.

It is important to acknowledge that while SCBEMs usher in a new area of research at the interface of reproductive and developmental biology, their aim is not to replace research with human embryos altogether. Instead, SCBEM research expands experimental horizons and possibilities to a degree that is not possible with human embryos. The substantial potential of these models can only be explored and developed with dedicated funding support and transdisciplinary collaboration. In providing this framework, we aim to stimulate these interactions through thoughtful research and responsible innovation, accelerating progress for the benefit of patients, families, and global public health.

## SCBEM: the state of the science and the need for standards

**Figure 1.**
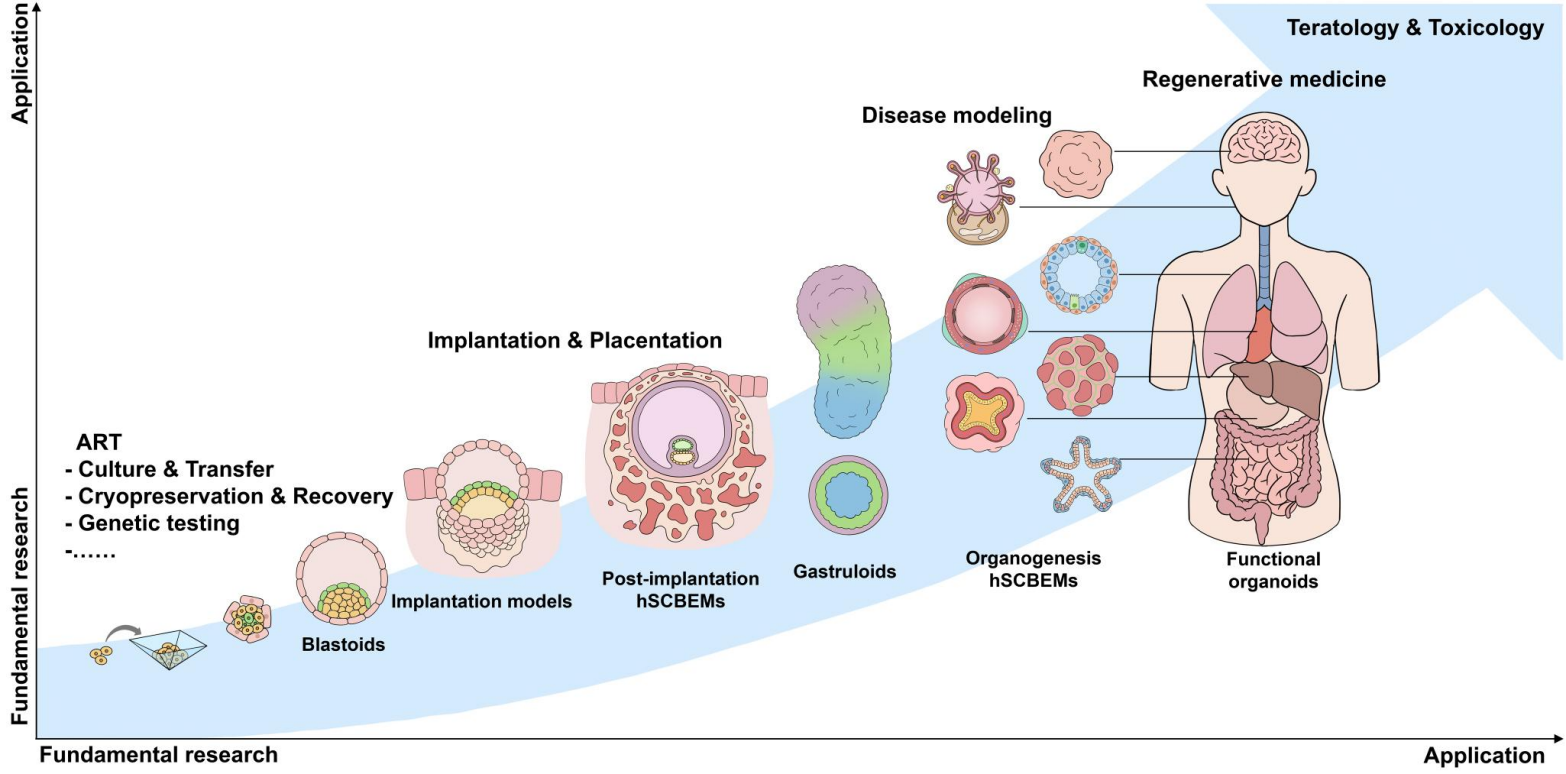
**Stem cell-based embryo models reflect different stages of human development**.

The development of SCBEMs stems from research on the self-organizing properties of multipotent and pluripotent stem cells (see Table 1), which allow the creation of organ models with minimal external inputs, e.g. intestinal and brain organoids (Sasai *et al*., 2012; Sasai, 2013; Kim *et al*., 2020). In contrast with these structures that represent defined differentiation paths leading to specific tissues or organs, SCBEMs mimic the whole or large parts of the embryo (Shahbazi, Siggia and Zernicka-Goetz, 2019; Fu *et al*., 2021; Turner and Martinez Arias, 2024) (Fig. 1).

At one end of the spectrum, research over the last few years with mouse PSCs and totipotent stem cells (TPSCs) (see Table 1) has yielded SCBEMs that mimic the development of a mouse embryo beyond gastrulation, up to an equivalent of 8.5 days from fertilization. These SCBEMs, albeit structurally defective, display beating primitive hearts and brain rudiments (Amadei *et al*., 2022; Tarazi *et al*., 2022; Li *et al*., 2025; Yilmaz *et al*., 2025). Nevertheless, these studies have revealed the remarkable potential of mouse PSCs and TPSCs. So far, similar work with human PSCs and TPSCs has not yielded structures beyond the earliest stages of gastrulation, Day 14 post fertilization, and those structures that emerge, do so at low frequencies and with variable morphology (Oldak *et al*., 2023; Chen *et al*., 2025). There are reports of human blastoids (see Table 1) being used as a starting point to trigger the development of a complete human embryo model, but the result, while containing features of a post-gastrulation embryo, is so far not comparable to the structures obtained in mouse models (Karvas *et al*., 2023; Liu *et al*., 2023; Oura *et al*., 2025). However, a recent report shows that monkey blastoids can be used to mimic gastrulation, though not organogenesis (Li *et al*., 2026). This suggests that in the future it might be possible to obtain similar structures with human PSCs. Such development would undoubtedly pose some ethical questions that need to be considered now.

In contrast with structures that display a complete body plan, the generation of human blastoids and gastruloids (see Table 1) is robust. Together, these SCBEMs span the first 4 weeks of the life of a human embryo. In the case of the pre-implantation period, research output on these structures has accelerated dramatically. Since the first reports of the development of a human blastoid, a number of peer-reviewed SCBEM protocols have been described (Liu *et al*., 2021; Yanagida *et al*., 2021; Yu *et al*., 2021; Kagawa *et al*., 2022). These blastoids reliably reconstruct all three blastocyst lineages with appropriate tissue-specific gene-expression patterns, albeit with a paucity of hypoblast cells in all cases.

Moving into the peri-gastrulation window, micro-patterned 2D colonies (Heemskerk and Warmflash, 2016) and 3D suspension gastruloids (Martinez Arias *et al*., 2022) diverge sharply in axial patterning. The former generates concentric germ-layer rings driven by gradients of signalling molecules. The latter break symmetry, extend a primitive-streak-like axis with little external intervention, and reconstruct axial organization with some associated derivatives, such as somites and a primitive heart.

This section summarizes the current situation in the field and highlights the steps needed to advance the field. We also survey the technical challenges the field faces and make recommendations to build a more harmonized and effective research ecosystem.

### Diversity of SCBEM protocols and developmental coverage

Recognition of the self-organizing abilities of PSCs and TPSCs has led to the development of the broad palette of SCBEMs outlined above. These structures are assembled from naïve, primed PSCs or, more recently, TPSCs corresponding to morulae stages. Each has its own characteristics, but all have been shown to be very sensitive to the initial state and culture conditions of the starting cell population. A clear example of the importance of the initial state of the PSCs and TPSCs for their appropriate differentiation has been provided by experiments aimed at obtaining a chimeric cynomolgus monkey PSCs (Cao *et al*., 2023). The pluripotency of human and the closely related NHP cells is generally tested in teratoma assays rather than in the gold-standard chimeric mouse assays (International Stem Cell Initiative, 2018). An exception is a recent study of chimerism with NHP (Cao *et al*., 2023). This work has shown that not all PSC starting populations have the same potential to be incorporated into a chimeric embryo or animal; culture conditions, passage number, origin of starting cells, and the reagents used in the pluripotency state crucially determine their potential to form chimeras. This experiment highlights significant challenges and bottlenecks for human SCBEM research that are reinforced by some experiments in the field (Oldak *et al*., 2023; Li *et al*., 2025; Yilmaz *et al*., 2025) and that need to be addressed.

Collectively, SCBEM protocols vary in their cellular origin (naïve vs lineage-primed PSC or TPSCs), culture methodology, aggregate size and surrounding geometry (U-bottom plates, AggreWell, stirred bioreactors), combinations of signalling molecules, matrix composition (Matrigel, Geltrex, laminin-521, or fully synthetic hydrogels), and external biomechanics (static, rocker, microfluidic perfusion). Such heterogeneity drives marked differences in the structural complexity of the SCBEM. Differences in germ-layer organization and proportions, polarity, growth, and developmental tempo make direct comparison challenging.

Presently, this diversity in SCBEM protocols often leads to variability in results across labs and difficulties in cross-referencing, which make it difficult to identify the source of divergent outcomes in the final structure. An important source of variability is differences between and within cell lines, reagents, and even operators. This is true even in the most robust models (e.g. blastoids and gastruloids) and becomes a challenge when dealing with many of the peri-implantation and peri-gastrulation models.

To make progress with SCBEMs, it is therefore essential to develop benchmarking through assays that allow categorization and selection of specific models. Both a general improvement and promulgation of standards for specific classes of models will be required (Martinez Arias *et al*., 2024). In the case of blastoids, for example, a reference has been established through a computational analysis that compares the different models with human IVF blastocysts in terms of gene expression and lineage representation (Zhao *et al*., 2025). Similar studies are being promoted with gastruloids (Balayo *et al*., 2025).

While SCBEMs have expanded our capacity to emulate the early stages of embryonic development, the field increasingly recognizes that technological sophistication alone does not guarantee interpretability. Notably, the International Society for Stem Cell Research (ISSCR)’s updated guidelines (Clark *et al*., 2025) call for delineating anchor points in embryo-like cultures that align with known *in vivo* developmental events, such as blastocyst cavity formation or the onset of gastrulation-specific gene expression. Given that SCBEM protocols often rely on a multitude of variables that can hamper reproducibility—even within the same laboratory—small differences in PSC lines, culture media batches, or operator handling can yield divergent results. These reproducibility challenges underscore the field’s pressing need for collective standardization.

### A call for standardization: methodological innovations and current constraints

Clear guidelines are indispensable for the development of SCBEMs, as emphasized in recent consensus-building efforts and opinion pieces where researchers propose criteria for monitoring their fidelity and efficiency (Martinez Arias *et al*., 2024). However, it is important to point out that while it may be relatively easy to standardize models derived from primed PSCs, since they are relatively genetically and epigenetically stable (Liu *et al*., 2017; Kilens *et al*., 2018), it appears to be more challenging to standardize naive PSCs as the current culture conditions for this state of pluripotency are suboptimal and extensive genetic and epigenetic aberrations accumulate upon extended culture (Pastor *et al*., 2016; Bar *et al*., 2017).

Suggested principles for standardization in the field include:

- **Use of reference cell lines:** a set of PSC lines (TSCs, ESCs, and iPSCs) characterized extensively at genetic, epigenetic, and functional levels, shared widely to serve as a common benchmark for SCBEM development; report pluripotency state, genetic integrity, and transposon status; use more than two lines per experiment.
- **Protocol transparency and harmonization:** standardize starting cell density induction modalities (e.g. small-molecule treatments, 3D aggregation parameters) and readouts (morphology, gene expression) to reduce experimental variability; disclose aggregation geometry, signalling protocols for use of small molecules or morphogens, matrix chemistry, and oxygen tension. Move toward open-source recipes to minimize reliance on proprietary, batch-variable commercial media.
- **Efficiency metrics**: state the percentage of aggregates that reach prespecified endpoints (e.g. more than 80% lumenized blastoids) and represent calculations in relation to the original number of starting aggregates on Day 0.
- **Fidelity metrics**: use of microfluidic culture, state-of-the-art microscopy techniques, and single-cell ‘omics’ to characterize lineages and enable real-time monitoring of cell fate decisions in SCBEMs and morphometrics to contemporaneous stages of *in vivo* human or NHP embryos.
- **Limitations and ethics**: declare missing tissues, off-target lineages, and provide an ethics statement covering embryo-likeness, data-sharing, and a description of the review of the experiment.

We suggest the development of a collective effort that identifies and characterizes at the genetic and epigenetic levels a small panel of reference PSC lines suitable for specific SCBEMs. It might well be that some cell lines will be particularly suitable for specific models. A similar effort should be made to standardize protocols, specifically in terms of initial culture conditions and the reagents used. It is a common experience that the reliance on commercial media has an underlying batch-to-batch variability that affects the experiments (Balayo *et al*., 2025). It might be necessary to work together with the companies that provide the culture media to develop the most reliable products that can advance the field. This collaboration would benefit both parties.

Standardized protocols and procedures to provide funders, policymakers, and the broader public with confidence in the rigour and reproducibility of embryo models, promoting transparency and informed engagement. One possibility is to establish a *Global SCBEM Reference Bank* housing genotyped, methylome-scored ESC/iPSC lines, paired with a public protocol repository, an inter-lab proficiency programme, and donor consent documentation.

### Limitations and barriers for progress

The main limitation of current models, particularly those that include primordia of several tissues and organs, is their reproducibility. Although useful in the present state for screens or modelling of the effect of specific mutations, these systems are highly sensitive to experimental conditions such as culture media, conditions, or the starting cell line. This is being addressed by in all cases, efforts are being made to enhance SCBEM fidelity through open discussions of methods across laboratories. Some specific recommendations to address this important issue are made below.

### Summary and recommendations

In summary, the diversity of SCBEM has enabled advancement but simultaneously complicates reproducibility, regulatory oversight, and progress towards translational goals. Altogether, SCBEMs provide a transformative and crucial resource to dissect the intricate choreography of early human development. As the field strives to balance ambition with responsibility, the future will likely witness further refinements in modelling, the establishment of consensus standards to ensure reproducibility, and more nuanced ethical frameworks that account for the fast-paced advancements of stem cell science. In response to these challenges, cohesive strategies are needed to ensure SCBEMs fulfil their scientific and medical potential responsibly:

- **Community-driven standardization and transparency.** Publication of protocols with detailed reagent lists, cell line specifications, and success metrics, emulating the organoid community’s best practices (Pasca *et al*., 2025), as well as fostering the development of open databases for SCBEM transcriptomic profiles, protocols, and troubleshooting tips. Additionally, the establishment of standards will be aided by rigorous comparison of human SCBEM data on pre-implantation IVF human embryos and NHP embryos, particularly during the peri-gastrulation stages.
- **Focused R&D to improve fidelity.** Develop better *in vitro* environments that recapitulate uterine cues or maternal factors, potentially via microfluidics or co-culture with endometrial cells or organoids.
- **Creation of Biobanks.** Biobanks should be created to distribute high-quality stem cell lines and organoids to researchers.

## Emergent technologies and applications

In the 1990–2000s, the discovery of human ESCs and iPSCs generated high expectations that these cells could be harnessed for disease modelling and to repair and replace a wide range of damaged organs and tissues. Some of these promises have been fulfilled (Robinton and Daley, 2012; Cyranoski, 2018)—most notably in the breakthroughs that have used PSCs to develop dopamine-producing neurones for Parkinson’s disease (Tabar *et al*., 2025), interneurones for epilepsy (Bershteyn *et al*., 2023), insulin-producing cells for Type I diabetes (Migliorini *et al*., 2021; Meng *et al*., 2025), retinal cells for a variety of retinal pathologies (Ahmed *et al*., 2021), and recently promising models of haematopoietic stem cells (HSCs) (Ng *et al*., 2025); some are now in advanced clinical trials. These individual successes show that high-quality discovery research can yield important biomedical breakthroughs with the potential to benefit patients (Adegunsoye *et al*., 2023).

Some of the challenges of using PSCs for regenerative medicine and disease modelling arise from the need for cells to be correctly organized in three dimensions. The development of organoids from adult stem cells (Kim *et al*., 2020) and brain and retinal organoids from PSCs (Sasai, 2013) has proven this point, but it is the emergence of SCBEMs that has opened the door to new technologies with potential applications in regenerative medicine and reproductive biology.

**Figure 2.**
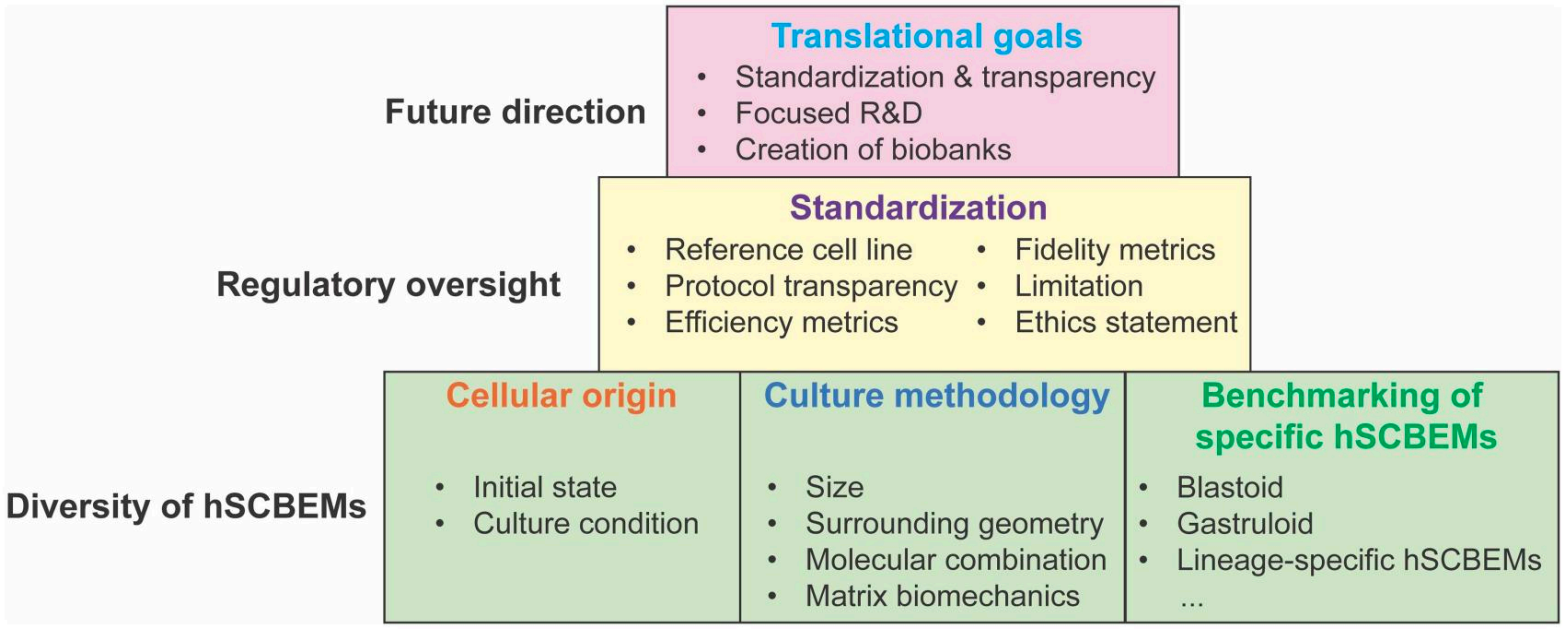
**Stem cell-based embryo models lend themselves to different applications in embryology, disease modelling, and toxicolog**y. R & D, Research and Development; hSCBEMs, human Stem Cell-based Embryo Models.

This section explores the potential technologies arising from SCBEM research and their application to specific challenges as well as their role in advancing training, method development, and safety testing (Fig. 2). While much of this discussion is forward-looking and acknowledges existing obstacles, we believe that now is the right time to outline the path from fundamental research to real-world impact.

### IVF and pre-implantation development

As discussed above, there is a rising demand for effective ART. Although there have been improvements success rates over the past several decades, it is still the case that only 30–40% of transferred embryos lead to a live birth, where maternal age plays a critical role (Wilcox *et al*., 2001; Li *et al*., 2015; Jarvis, 2016; Human Fertilisation and Embryology Authority UK, 2025). Presently, fertility clinics participate in large-scale studies aimed at understanding the basis for these success rates and developing approaches to improve them (Coticchio *et al*., 2025).

The first major challenge in ART arises after fertilization, at the eight-cell stage, when most embryos arrest due to aneuploidies that are a consequence of meiotic or mitotic errors. Currently, human embryos are the only source of experimental material to study this very early developmental phase in humans. However, researchers are working to develop cultures of TPSC that mimic four-cell and eight-cell embryos (Li *et al*., 2023) to use them in embryo models in mouse (Li *et al*. 2025; Yilmaz *et al*., 2025). However, it is early days. In the future, another source of models for research on these early stages will be embryos derived from *in vitro* gametogenesis (see Table 1) (Saitou and Hayashi, 2021).

A second barrier to successful ART is the implantation of the blastocyst into the uterus, which, though dependent on negotiating earlier stages successfully, might be associated with physiology rather than the genetics of the blastocyst, including its compatibility with the ‘endometrium’ (see Table 1). Here, blastoids are a promising model; even though they are not the result of fertilization and cleavage, they nevertheless exhibit sufficient similarities at the level of gene expression and cellular organization to the blastocyst (Zhao *et al*., 2025) to warrant their use to study cryopreservation and culture conditions for blastocyst competency as well as the initial stages of implantation; they can also be used in teaching.

The ability to produce large numbers of blastoids means that they could provide a faithful substrate at scale to test for compounds that can improve blastocyst health and viability. They could also be used to support training in clinics, to develop improved techniques for embryo freezing and recovery, and to test the quality of reagents such as culture media before they are applied to embryos. Collaborative efforts are underway between researchers and IVF clinics to determine whether blastoids show measurable sensitivity to conditions that affect embryos as a means to determine whether blastoids could provide a surrogate model for quality control in ART.

### Implantation and early placentation

Successful human development depends critically on the establishment of structural and functional connections between the conceptus and the endometrial lining of the maternal uterus (Bondarenko and Turco, 2025). The process is initiated by the attachment of the blastocyst to the endometrium, followed by its deep implantation into the uterus. Completion of embryo implantation frequently fails in both assisted and unassisted pregnancies for reasons that are poorly understood and difficult to study *in vivo*.

Once a blastocyst has implanted, early events lead to the establishment of the maternal–foetal interface and to the initial formation of the placenta, which is the organ that mediates nutrient, gas, and signalling exchange between maternal and foetal systems later in pregnancy. Defects in interactions between conceptus (see Table 1) and endometrium and in early placental development underlie major pregnancy disorders, including miscarriages, foetal growth restriction, and pre-eclampsia (Smith, 2010). Understanding how embryonic and extraembryonic layers (see Table 1), e.g. placenta and uterine compartments, coordinate to establish these interactions is critical for uncovering the mechanisms that drive healthy pregnancy outcomes. Having *in vitro* models of human blastocyst attachment, implantation, and invasion is an important aim to improve ART and to understand the early origins of common pregnancy conditions.

SCBEMs, including blastoids, are, in principle, suitable for use in research to address some of these issues. Although mouse and primate blastoids have not yet led to a successful implantation in animals, blastoids are able to recapitulate the early stages of the process ([Bibr deag035-B92][Bibr deag035-B93]; Li *et al*., 2023).

Human blastocysts and blastoids can attach and implant *in vitro* into endometrial models that recapitulate some features of receptive endometrial tissue. In these conditions, both blastocysts and blastoids form embryonic cell types as well as extraembryonic derivatives fated to produce early placental cells, which contact the underlying endometrial cells. These systems are complemented by single-tissue cultures, including trophoblast stem cell derivatives established from blastocysts, PSCs, or placental tissues, which extend approaches that can investigate post-implantation placental development (Turco *et al*., 2018; Dong *et al*., 2020).

Methods to mimic the endometrium *in vitro* include 2-dimensional and 3-dimensional models that are typically formed from endometrial stromal and epithelial cells (Bondarenko and Turco, 2025). Early models of implantation enabled the study of embryo and blastoid attachment and initial trophoblast invasion (Boretto *et al*., 2017; Turco *et al*., 2017; Ruane *et al*., 2022; Shibata *et al*., 2024). Recent developments have improved the complexity and physiological relatedness of endometrial models and have been able to elicit processes mimicking implantation using blastocysts and blastoids (Song *et al*., 2026; Li *et al*., 2025; Mole *et al*., 2026). These systems promote investigation of the early maternal–foetal interface and identification of molecular pathways through which cells of the conceptus and endometrium interact. Patient-derived endometrial organoids were also used to run a proof-of-concept screen for drugs that might affect these interactions and influence Recurrent Implantation Failure (Li *et al*., 2025). While valuable, existing endometrial and implantation models still lack the cellular complexity, specifically endothelial and immune cell populations found *in vivo*. This structural discrepancy highlights the need for careful interpretation of *in vitro* findings.

Challenges remain, as this period of development is very difficult to access *in vivo*, and consequently, there are limited data with which to benchmark the *in vitro* systems. Moving forward, careful synchronization of endometrial, placental, and embryonic models, aligned to equivalent gestational stages and supported by *in vivo* criteria where possible to assess model fidelity and functional integration, will be essential to advance *in vitro* studies of human development and the maternal–foetal interface. A future step for these models will be to achieve a degree of vascularization, which will be beneficial for sustaining growth and supporting physiologically relevant functions of the SCBEMs. These advanced models will be crucial to exploring the complex and dynamic conceptus–maternal interactions and help address key biological questions around early human development, with important implications for improving IVF success and obstetrical outcomes.

### Disease modelling

The advent of iPSCs has opened up the possibility of modelling disease by obtaining iPSCs from patients and differentiating them along specific lineages (Rowe and Daley, 2019). This has been done with some success in the case of PSC-derived neuronal (Velasco *et al*., 2020; Sidhaye and Knoblich, 2021) and intestinal (McCracken *et al*., 2014; Aurora and Spence, 2016) organoids. However, these systems lack the integration of components from different germ layers and, in particular, innervation and an immune environment that have to be added through the composition of what are called assembloids—*in vitro* models that combine two or more organoids (Miura *et al*., 2022). SCBEMs have the potential to provide this ‘for free’ due to their integrated nature. Some gastruloid derivatives have already been used in this manner to show proof of principle studies of an infant leukaemia in the blood (Ragusa *et al*., 2025) and of skeletal pathologies such as spondylocostal dysostosis (Yamanaka *et al*., 2023).

There are strong possibilities in the context of cardiac development, as the heart is an organ assembled from contributions of several lineages that come together naturally in SCBEMs. Furthermore, the modularity of the system and the possibility of assembly at a different level allow the study of the role that cell interactions play in the development of syndromes and disease.

While it is early days, the potential for SCBEMs to assist in disease modelling is clear and, together with some of the developments for pre-implantation and implantation diagnostics, is likely to be one of the most beneficial applications of SCBEMs.

### Regenerative medicine

An important aim of research with PSCs, yet one where PSC-based approaches have not been as successful as expected, has been the development of tissues and organs to replace damaged ones. However, it is in this area that, in the long term, SCBEMs are likely to have an impact because their recapitulation of all important events of gastrulation leads to more faithful primordia for tissues and organs.

The blood system is a good example of an organ with a high unmet clinical need that might be attainable using SCBEM systems. Blood diseases are a major cause of problems in the form of birth defects and cancer. Over the last 50 years, the solution to these problems has been bone marrow transplantation, which carries both the challenge of finding immunological matches for transplantation and a significant risk of serious complications, including graft-versus-host disease. iPSCs provide an alternative, but they require differentiation into HSCs. While progress has been made in this direction, the formation of HSCs requires interactions between different tissues and the generation of a niche, which are not easily reproducible *ex vivo*. Some progress has been achieved in the last years, but the protocols are complex and difficult to reproduce because of the difficulty of substituting signalling cocktails for *in vivo* interactions. It is here that, as in other cases, the ability of SCBEMs to autonomously mimic the necessary interactions that happen in the embryo harbours some promise.

### Teratology and toxicology

The effect of teratogens on the development of the embryo is a major source of concern for the health of the pregnant person and the newborn. There is evidence that several compounds have effects early in development, where the embryo is most vulnerable to chemical exposure. Animal and cell-based models have been developed over the years to configure a palette of systems used by the industry (Carter, 2020). However, there is a lack of proper models that reflect human development. This lack has had consequences in the past, as shown in the case of thalidomide, where the response in human development was not predicted from testing with animal models (Vargesson, 2015). SCBEMs have the potential to fill this gap and play an important role in delivering animal free, human-specific systems for drug and teratogen testing (Marikawa, 2022). However, as stressed elsewhere, SCBEMs should be seen and used as an extremely valuable resource that reduces, but does not substitute, animal testing.

In principle, blastoids, gastruloids, and related SCBEMs can be used as substrates to test the effects of potential teratogens on the early stages of development. While some of this work can be done on single-tissue cellular models, e.g. cardiomyocytes, these studies miss the interactions between distinct lineages that are the basis of tissue and organ formation. Gastruloids have already proven to be good substrates for these tests, as they model many of the features and processes associated with germ-layer organization and gastrulation movements that are associated with the action of teratogens (Mantziou *et al*., 2021; Marikawa, 2022; Huntsman *et al*., 2024). Blastoids can also be used in this manner, with implantation models playing an important role as a functional test (Zhang *et al*., 2023; Liu *et al*., 2025).

An interaction between the SCBEM and the teratology research communities would be a profitable way to not only develop the models further by answering the demands and standards of the field but, more significantly, to provide a much-needed model closer to the embryo than any model currently available.

### Limitations and barriers to technology development

Despite their promise, current SCBEMs require further development. If they are going to fulfil their promise and the needs stated above, these developments need to be focused not only in terms of the complexity of the models but also the technical aspects of their robustness, production, and quality control. SCBEMs also require further validation against *in vivo* tissues and embryos, where possible. Furthermore, it is important to acknowledge that the current cadre of SCBEMs is unsuitable to understand most causes of IVF failures that relate to errors in fertilization or the divisions leading to the morula. Consequently, and in particular for these early stages, research with human embryos will continue to be an essential source of information.

An important current challenge to the use of SCBEMs in reproductive biology is the lack of a faithful uterine model that fully mimics the maternal environment that provides metabolic processing. While closing this gap, ongoing work using organoids and SCBEMs is revealing how much is autonomous in the embryo and the endometrium and, in this manner, such work is expanding our understanding of the deep biology of the reproductive system.

### Summary and recommendations

In summary, SCBEMs have the potential to advance our understanding of reproductive biology and associated technologies. Here, we would like to make some recommendations that should be taken into consideration during the technological development of the field.

- **Design specialized SCBEM platforms for infertility research.** This will require optimizing IVF protocols, understanding implantation failures, and teratogenicity testing (screening pharmaceuticals or environmental agents).
- **Promote interactions between SCBEM researchers and the ART community**. This should be developed and facilitated, as clinical needs will strengthen the research field and focus the applications that arise from it. For instance, it should be established whether SCBEMs are sensitive to conditions such as poor-quality media or teratogens that affect embryo development, i.e. it should be determined to what extent SCBEMs can provide a meaningful surrogate.
- **The development of high standard, clinical grade models**. This should be set up to serve as a basis for the practical applications of SCBEMs, particularly those associated with teratology and regenerative medicine.
- **Build bridges to clinical applications.** Promote interactions between the private and academic sectors in the design of specialized SCBEM platforms to optimize IVF protocols, understand implantation failures, and teratogenicity testing (screening pharmaceuticals or environmental agents). Integrate patient-specific iPSC lines to identify individual genetic or epigenetic risk factors for congenital anomalies or early pregnancy loss.
- **Development and support of training workshops.** To encourage the uptake of SCBEMs in applied research in IVF and teratology as widely as possible.

## Ethical and legal considerations of SCBEMs

Research involving SCBEMs raises some specific ethical issues and requires an updated international ethical framework that builds on existing guidelines for research with human stem cells and embryos while accounting for SCBEMs’ unique characteristics. Below, we provide an overview of the ethical and legal considerations associated with SCBEMs and a summary of existing international guidelines and ongoing discussions.

### Ethical considerations

SCBEMs differ in ethically significant ways from human embryos used in ART that are sometimes a reference in ethical discussions. First, SCBEMs are not formed by the direct fusion of egg and sperm and, unlike embryos that are intended for parental projects, they do not have intended parents. Instead, they are generated from donated cells. Second, SCBEMs are not created with the intent to be transferred to, or gestated in, a human body or *ex vivo* through ectogenesis. The scientific and medical research using SCBEMs is therefore decoupled from the ethical issues related to parenthood and pregnancy, and the associated impact on the pregnant person’s bodily autonomy. The ethical justification for creating SCBEMs lies in their intended purpose: they are generated to advance scientific and medical knowledge and to offer an ethical alternative to the use of human embryos and nonhuman animals in research (Rivron *et al*., 2018a,b).

SCBEM research shares many ethical considerations with conventional stem cell and embryo research. These include ensuring informed consent from donors of biological material, clarifying the scope of applications for the cells and tissues generated and the scope of intellectual property rights (e.g. patents and licenses), establishing effective oversight and governance, and determining how to inform the public of advances in the field and where and when to involve them in deliberations around what limits, if any, should be imposed on research. Many of these concerns may be addressed through established ethical frameworks for ESCs and iPSCs and, to some extent, existing public engagement strategies.

Additional ethical challenges are specifically posed by SCBEMs because they are explicitly designed to replicate aspects of human embryonic development, either partially or in full. SCBEMs thus have specific ethical implications depending on how closely they mimic human embryos, including their degree of structural completeness and the appearance of features of concern (Hyun *et al*., 2020; Clark *et al*., 2021; Lovell-Badge *et al*., 2021; Moris *et al*., 2026).

An important concern arises from their developmental potential. Currently, their progress is limited to a few days and cannot form viable foetuses (Rivron *et al*., 2018a,b; Li *et al*. 2023). Furthermore, if a SCBEM lacks essential cellular components (such as the ones normally fulfilling the functions of the placenta) or if it has significant genetic and epigenetic abnormalities (such as acquired genetic changes that would be incompatible with continued development—a prominent phenomenon in *in vitro* culture), the model will not possess the inherent capacity to develop into a viable foetus. Such biological constraints may ease certain ethical concerns, but not all. For example, there is a possibility that SCBEMs could be used to form part of foetuses. Addressing this issue requires careful consideration of the actual developmental potential of specific human SCBEMs.

A second category of concern relates less to developmental potential and more to their symbolic value: the ethical unease that arises from the creation and destruction of entities expressing features commonly considered to define early human life and identity. These features may include the formation of a hand, a face, or a beating heart. While the moral relevance of symbolic value remains a subject of ethical debate (de Graeff and De Proost, 2025), the possibility of restricting SCBEMs from developing certain tissues or progressing through certain stages of development may reduce objections in those who harbour these concerns.

A third type of concern relates to the potential development of SCBEMs with an integrated central nervous system with sentience or consciousness as it occurs at late stages of foetal development. Here, the concerns shift to the respect owed to sentient and/or conscious beings and the imperative not to harm them.

Taken together, these specific concerns associated with SCBEMs highlight the importance of carefully defining their biological capabilities and setting limits relative to the emergence of features of moral concern.

### Legal considerations

The emergence of SCBEMs, and their increasing complexity, raises questions around how this research should be regulated. Existing national or regional laws governing human embryo research may apply depending on how a human embryo is legally defined. Some SCBEMs may legally qualify as human embryos in certain jurisdictions—either due to ambiguous legal definitions or because they meet existing legal criteria, e.g. Australia (Matthews and Morali, 2020; Fabbri *et al*., 2023). Alternatively, SCBEM research could be regulated separately to embryos (Agence de la biomédecine, 2023; Nuffield Council on Bioethics, 2024; Statens medicinsk-etiska råd, 2024; Cave, 2025) or embryo research laws could be reformed to provide oversight through existing embryo research governance frameworks. Researchers and funders must be alert to how SCBEMs are classified in their jurisdictions, as embryo definitions vary widely and may need updating to address emerging scientific realities and community concerns.

In addition, jurisdictions should seek to establish limits or boundaries where SCBEM research is not already captured under existing laws and regulations. For example, practices such as transferring human SCBEMs into the uterus of a living human or animal, or attempting to bring them to viability through ectogenesis, are widely regarded as unethical, unjustified, and unsafe. These actions should be explicitly prohibited under law to ensure responsible scientific conduct (Lovell-Badge *et al*., 2021; Clark *et al*., 2025).

Importantly, the purposes of legal boundaries include fostering and safeguarding safe, ethical, and responsible research. These limits serve to assure the public that scientific progress is pursued with ethical restraint and respect for moral pluralism. The 14-day rule exemplifies this function of regulatory limits—not because it resolved all ethical disagreements, but because it demonstrated deference to diverse moral views (Hyun *et al*., 2016). Ongoing discussions about extending this limit to 28 days—allowing researchers to culture human embryos longer to explore otherwise inaccessible stages of development—are highly relevant to the evolving context of SCBEM research. As scientific capabilities advance, similar regulatory boundaries may be necessary to maintain public trust and accountability. The recent suggestion of a 28-day rule for human blastoids—i.e. allowing the culture of blastoids to a structural equivalent of 28 days—by ethics committees in France and the same proposition by Swedish (Statens medicinsk-etiska råd, 2024) and Dutch (Health Council of the Netherlands, 2023) Councils demonstrates how regulation can adapt to evolving science while fostering innovation and safeguarding public confidence.

### International guidelines

In light of the ethical and legal considerations, several groups have held discussions and developed guidelines and recommendations. Two major scientific societies—the ISSCR (International Society for Stem Cell Research, 2025) and the European Society for Human Reproduction and Embryology (ESHRE) (Popovic *et al*., 2019)—have led this discussion, with others such as the Nuffield Council on Bioethics (Nuffield Council on Bioethics, 2024) and the UK Cambridge Reproduction and Progress Educational Trust (Cambridge center for Reproduction, 2024) also providing guidance. Recommendations of the ISSCR have been adapted and implemented at the national level by France (Agence de la biomédecine, 2023) and propositions were made in Sweden (Statens medicinsk-etiska råd, 2024), the Netherlands (Health Council of the Netherlands, 2023), and the UK (Nuffield Council on Bioethics, 2024). These guidelines and recommendations will need to be updated regularly as scientific progress is made, as is illustrated by a recent proposal to revise the ISSCR 2021 Guidelines (Clark *et al*., 2025). While guidelines and recommendations inevitably vary based on jurisdictions, various core areas of consensus for the ethical oversight of SCBEM research can be identified:

- Due to their limited developmental potential, SCBEMs in their current state should not be considered human embryos in either a biological or legal sense (Cave, 2025). This is the position in most countries, but, notably, this position is not reflected in the Australian legislation (National Health and Medical Research Council, 2023) and in a proposal by the Dutch Health Council to revise the Dutch Embryo Act (Health Council of the Netherlands, 2023; M’Hamdi and de Wert, 2024), which both consider certain SCBEMs equivalent to human embryos.
- Research involving 3-dimensional SCBEMs requires ethical oversight by competent authorities, preferably ethics committees experienced in overseeing research with human embryos (Clark *et al*., 2025; International Society for Stem Cell Research, 2025).
- Practices that are currently unjustified, unethical, or unsafe should be prohibited. These include the transfer of human SCBEMs into the uterus of a living human or animal and attempts to bring a human SCBEM to viability through ectogestation (Hyun *et al*., 2020; Clark *et al*., 2021, 2025; Lovell-Badge *et al*., 2021).
- Scientific societies and ethics committees should ensure that the *in vitro* development of human SCBEMs happens incrementally and that the quality and reproducibility of results are assured and shared before researchers explore later developmental stages. A gradual pace and a focus on quality and reproducibility help justify the research, limit the likelihood of precipitous research in areas of widespread ethical concern without broad consultation, and allow assessment of whether societal benefits can be achieved. Some criteria for the characterization and evaluation of SCBEMs have been described (Posfai *et al*., 2021; Martinez Arias *et al*., 2024).
- SCBEMs should be maintained in culture for the minimum time necessary to achieve each study’s scientific and/or medical objective(s). Permission to culture should take into account the quality of the model, the justification of the objectives, the technical feasibility, and the consistency of the practice with local laws and international guidelines (Lovell-Badge *et al*., 2021; Rivron *et al*., 2023a,b).
- The extent to which a model is suitable for answering a specific research question should guide ethical oversight (Jonlin *et al*., 2025). Forming an SCBEM that is more complete than necessary for a particular study might create unnecessary ethical and legal concerns. Therefore, for each specific research endeavour, less complete models should be preferred (Rivron *et al*., 2023a,b). While the use of more complete SCBEMs may be especially useful to research the early stages of human development, where possible, less complete models or other alternatives (e.g. organoids) should be preferred for studies addressing later stages of human development (Rivron *et al*., 2023a,b).
- Permission from an ethics committee to culture human SCBEMs that are more complete or complex, reflecting an increasing potential for prolonged integrated development or accumulating additional features of concern, requires a proportional increase in potential benefits.
- The benefits of SCBEM research should outweigh the ethical risks and concerns associated with them. Specifically, SCBEMs that contain not only the embryonic but also the extraembryonic tissues (or support element fulfilling extraembryonic functions) that control embryo/foetal development and enable implantation in the uterus should require a significant proportional increase in potential benefits, as these SCBEMs may, with further technical advances, support the development of a foetus (Clark *et al*., 2021).
- Although SCBEMs are useful for research and provide opportunities for further scientific advances, the limitations of current scientific knowledge and regulatory constraints must be clearly explained in any communication with the public or media (Rivron *et al*., 2023a,b). Because this area of science moves quickly, international guidelines need to be regularly updated. The following three issues are currently under discussion.

### Could ethical oversight be graded according to an international SCBEM classification?

Categorizing SCBEMs based on their developmental capacity has been suggested as one way to frame ethical concerns and to enable adequate ethical oversight. A distinction was initially drawn between integrated and non-integrated SCBEMs. Integrated SCBEMs comprise both embryonic and supporting extraembryonic tissues—such as blastoids—and may, when improved, acquire the capacity to develop into a foetus. Non-integrated SCBEMs—such as gastruloids—form anatomical structures that may support the development of integrated organs but so far lack the potential to generate a full foetus (Clark *et al*., 2021). However, in the face of the proliferation of different models, the ISSCR has recommended that the use of this distinction to define ethical oversight be discarded and that all SCBEM research undergo a specific ethical and scientific oversight process, graded according to the complexity of the model. Improving rather than abandoning this classification has been proposed (Ismaili, 2025). This classification could, for instance, be based on the inferred capacity to support the development of a foetus or the appearance of features of moral value, including the emergence of structures that could give rise to sentience and consciousness. This enhanced ethical oversight may require additional support for ethics committees, for example:

- by creating a register of decisions made by different ethics committees and the decision-making process, including the rationale for each type of SCBEM, which may help other committees,
- by establishing an international group of advisory ethicists to assist other committees in decision-making,
- by providing guidance, for instance, through international recommendations, on what time limits and/or developmental boundaries should be placed on SCBEMs.

Research with SCBEMs—particularly the most complete models—is not regulated by default in most countries and therefore not limited in time. As previously mentioned, an exemption to this is Australia, where the culture of human blastoids is limited to 14 days, as they are deemed to be legally equivalent to embryos. In France, the culture of integrated SCBEMs is permitted up to a developmental stage equivalent to 28 days of a human embryo, and—as previously mentioned—similar recommendations have been made by Dutch and Swedish councils. These developmental limits are intended to allow research that sheds light on the so-called ‘black box’ period of human development, from Day 14 to the beginning of the second month. This 28-day limit is being proposed to ensure a gradual and justified use of these models while signalling to the public that research is not uncontrolled. It still limits some opportunities to study developmental events that are important for human health and may, in turn, be debated again as science progresses. As development progresses, SCBEMs often progressively and significantly deviate from natural development and thus lose scientific and medical relevance, making them scientifically less suitable and potentially ethically less acceptable. Although the stage of 28 days, Carnegie stage 10, has not been achieved to date, with advancing technologies such as better stem cells and supporting endometrial organoids, bioreactors, and oxygenation systems, culturing SCBEMs to this stage might become technically feasible and scientifically and medically valuable in the future. At 28 days, the nervous system is underdeveloped, alleviating ethical concerns related to sentience.

The Nuffield Council on Bioethics report, like the UK Code of Practice, recommended a case-by-case assessment but included a backstop of SCBEM development to an equivalent stage of a 56-day embryo. Importantly, after the first 2 months, alternative models—such as non-integrated SCBEMs focused on specific organs, organoids, or assemblies of organoids—can serve many research purposes, rendering continued use of integrated SCBEMs less necessary and potentially unjustified. Although there is broad agreement on the need to limit SCBEMs’ culture time, there is no international consensus on the specific threshold. Disagreements stem from a lack of shared criteria for setting such limits and the difficulty of applying rigid policies to complex and evolving technologies. An alternative or complementary regulatory approach focuses on monitoring ethically significant developmental features as a trigger for enhanced oversight, rather than relying solely on fixed time-based boundaries.

### What if advances make SCBEM indistinguishable from human embryos?

If human SCBEMs approach biological similarity with human embryos, it is likely that there will be increasing calls for the legal definition of a human embryo to be amended such that research using relevant models would be subjected to similar stringent ethical and legal protections. Any updated definition should consider that SCBEMs that do not reflect the complete embryo might, nevertheless, be able to form a foetus. Since technical solutions have been developed to fulfil the extraembryonic, uterine, and physiological functions that support the development of a foetus (e.g. hydrogels, bioreactors), a refined legal definition should—where relevant—take into account not only the intrinsic potential of the cells to form a foetus but also their dependence on essential external support functions that aid development.

Given that transferring human SCBEMs into a uterus to determine developmental potential is prohibited and considered unethical (Clark *et al*., 2025), alternative strategies to evaluate such potential—sometimes referred to as Turing Tests (Rivron *et al*., 2023a)—have been proposed. According to one of the proposed approaches, a SCBEM could warrant treatment equivalent to that of a human embryo if it can both accurately replicate embryonic development up to a certain stage deemed to be the ethically acceptable limit and demonstrate the capacity to generate living, fertile organisms in multiple species, especially NHPs. Reaching this dual threshold may suggest that the SCBEM warrants a legal treatment like that of a human embryo under ethical and regulatory frameworks (Rivron *et al*., 2023a).

### Public transparency and communication

Alongside considering the ethical and regulatory implications of SCBEMs and how these may evolve as the science advances, there is a strong need for transparent and clear public communication of this research and its potential impact on human health and medical research and its limitations (Rivron *et al*., 2023b). Given the resemblance of SCBEMs to human embryos, even if in limited ways, they attract public and media interest, including in the EU where human embryo research is a matter of public concern (Pucelj and Matusiak-Frącczak, 2024).

Although SCBEMs may provide a more ethically acceptable alternative to the use of human embryos and animals for research, exactly what SCBEM research entails, and why such research is being undertaken, may not be immediately clear to people in the broader community. Thus, there is an ongoing need for openness and public engagement to learn more about which aspects of SCBEM research and application are important to individuals and why, and what steps could be taken to reassure or alleviate concerns and questions they may have. Conversely, a lack of transparency about SCBEM research and its objectives, overstating achievements, potential benefits, or failure to acknowledge and respond to community concerns, risks eroding confidence in science and ultimately public trust (Lovell-Badge *et al*., 2021; Rivron *et al*., 2023b).

In their public communication, researchers must be clear about the similarities and differences between SCBEMs and human embryos, in particular their inability or limited ability to develop through different stages, form features of moral value, and acquire sentience and consciousness. They should also clearly explain the scientific and/or medical purpose of the research. Public debates around embryonic stem cell research have shown that many people consider the research’s purpose—either scientific or medical—to be important when assessing research acceptability. SCBEM research both advances basic knowledge of human development and enables research to improve treatments for infertility, pregnancy loss, congenital or environmentally induced birth defects, and subsequent adult diseases. Clear communication about these goals in lay terms can help members of the public gain insight, can more fully involve members of the public in ongoing discussions, and can provide opportunities for them to share their views on ethical considerations and what limits, if any, should be imposed by regulators, considering the potential scientific and medical benefits.

As part of a transparent process, a public register of laboratories involved in SCBEM research could be useful (Nuffield Council on Bioethics, 2024). To foster public dialogue, researchers, regulators, and funders will need to proactively consider the ethical aspects of SCBEM research, develop clear and well-reasoned explanations for their studies, and be able to communicate these in a timely and accessible manner (Lovell-Badge *et al*. 2021; Rivron *et al*., 2023a,b). They will also need to engage members of the public to hear their hopes, fears, enthusiasm and concerns about SCBEM research, and the reasons for their views and facilitate wide representation in public engagement.

Groups in two countries have recently begun to engage the public in discussions about SCBEM research. In the Netherlands, lay participants with no prior knowledge about this type of research requested to be more informed about the research and involved in its regulation (Pereira Daoud *et al*., 2024). Moreover, additional dialogues with the lay public are underway (Rathenau Institute, 2025). In the UK, a series of facilitated dialogue events between members of the public who had some prior knowledge of embryo research, with scientists, legal experts, and ethicists, demonstrated that most participants viewed SCBEMs as different from embryos and that uncertainties about the potential uses of SCBEMs raised specific concerns (van Mil, 2024). There was also a call for regulation of SCBEM research in some form and the involvement of public voices in this to give a broad range of perspectives, which are recommendations incorporated into the UK Code of Practice.

In sum, future public engagement efforts need to extend beyond just telling people about SCBEM research and its potential implications and involve careful planning, dedicated resources, and the expertise of public engagement and dialogue specialists. Researchers, institutions, regulators, and funders need to coordinate efforts and work with other interested members of the community to consider how to best foster public discourse and involvement. In addition, this research and its ethics should also be discussed in the classroom.

### Summary and recommendations

The development of SCBEMs cannot happen without ethical and legal oversight and proper communication of its findings with the public, as well as with funding and governmental institutions. As a summary of the discussion on this section, here are some recommendations in this area for the field.

Establish a distinct legal and ethical status for SCBEMs

- Due to their limited developmental potential, SCBEMs in their current state should not be considered human embryos in either a biological or legal sense.
- Explicitly prohibit the transfer of human SCBEMs into the uterus of any living animal or human and attempts to achieve ectogenesis (the development of an organism to term outside a biological womb).

Address key ethical and regulatory challenges

- Ensure the implementation of international ethical guidelines, such as those developed by the ISSCR and ESHRE, across the world.
- Ensure oversight of SCBEM research, particularly regarding the formation of features of ethical concern, including the potential emergence of sentience.
- Support ethics committees by establishing an open-access register of decisions taken by the different ethics committees along with the decision-making process, including the rationale for each type of SCBEM, and by setting up an international group of advisory ethicists to assist other committees in decision-making.
- Mandate informed consent from donors of biological material used in SCBEM research.

Adopt guidelines and oversight adapted to complexity

- Encourage incremental research approaches, with limits on culture durations and proportional justification for more complex SCBEM models.
- Design oversight mechanisms that adapt to the increasing sophistication of SCBEMs and advances in science.
- Identify, introduce, and monitor specific boundaries, including time limits on culture (e.g. France’s 28-day proposed limit), while aligning with international standards.

Foster public trust through transparency and communication

- Promote transparent and accurate communication about the limitations, purposes, and safeguards of SCBEM research.
- Engage citizens and stakeholders in ongoing dialogue about acceptable ethical boundaries, building public trust in the governance of SCBEMs.
- Engage academic institutions/universities to promote the ethical and regulatory discussions when SCBEM are object of lectures.

Implement dynamic governance frameworks

- Require regular updates to SCBEM guidelines as scientific capabilities evolve.
- Identify developmental features of ethical concern and ensure these remain at the core of public accountability strategies.

## Data Availability

There is no specific data generated for this article. The data in support of the statements made are available in the publications that are quoted associated with it.

## References

[deag035-B1] Abas R , Masrudin SS, Harun AM, Omar NS. Gastrulation and body axes formation: a molecular concept and its clinical correlates. Malays J Med Sci 2022;29:6–14. 10.21315/mjms2022.29.6.2 PMC9910376 36818899

[deag035-B2] Adegunsoye A , Gonzales NM, Gilad Y. Induced pluripotent stem cells in disease biology and the evidence for their *in vitro* utility. Annu Rev Genet 2023;57:341–360. 37708421 10.1146/annurev-genet-022123-090319

[deag035-B3] Agence de la biomédecine. *Biomedicine Act: Opinion of the Conseil d’orientation on Stem Cell-Based Embryo Models*. 2023. https://back.agence-biomedecine.fr/uploads/22_06_avis_du_co_embryoi_des_eng_2_9bd67f7b22.pdf (6 February 2026, date last accessed).

[deag035-B4] Ahmed I , Johnston RJ Jr, Singh MS. Pluripotent stem cell therapy for retinal diseases. Ann Transl Med 2021;9:1279. 34532416 10.21037/atm-20-4747 PMC8421932

[deag035-B5] Alves-Pimenta S , Colaco B, Oliveira PA, Venancio C. Development features on the selection of animal models for teratogenic testing. Methods Mol Biol 2024;2753:67–104. 38285334 10.1007/978-1-0716-3625-1_3

[deag035-B6] Amadei G , Handford CE, Qiu C, De Jonghe J, Greenfeld H, Tran M, Martin BK, Chen DY, Aguilera-Castrejon A, Hanna JH et al Embryo model completes gastrulation to neurulation and organogenesis. Nature 2022;610:143–153. 36007540 10.1038/s41586-022-05246-3 PMC9534772

[deag035-B7] Aurora M , Spence JR. hPSC-derived lung and intestinal organoids as models of human fetal tissue. Dev Biol 2016;420:230–238. 27287882 10.1016/j.ydbio.2016.06.006 PMC5140713

[deag035-B8] Bai Z , Han J, An J, Wang H, Du X, Yang Z, Mo X. The global, regional, and national patterns of change in the burden of congenital birth defects, 1990-2021: an analysis of the global burden of disease study 2021 and forecast to 2040. EClinicalMedicine 2024;77:102873. 39416384 10.1016/j.eclinm.2024.102873 PMC11474384

[deag035-B9] Balayo T , Lunn S, Pacual-Mas P, Fiuza U, Vasudevan A, Frenster J, Galloon HY, Flores Peiras R, Martinez Arias A, Dias A et al N2B27 media formulations influence gastruloid development. bioRxiv. doi: 10.1101/2025.03.15.643474v1, 2025, preprint: not peer reviewed. PMC12687332 41287934

[deag035-B10] Bar S , Schachter M, Eldar-Geva T, Benvenisty N. Large-scale analysis of loss of imprinting in human pluripotent stem cells. Cell Rep 2017;19:957–968. 28467909 10.1016/j.celrep.2017.04.020

[deag035-B11] Bershteyn M , Broer S, Parekh M, Maury Y, Havlicek S, Kriks S, Fuentealba L, Lee S, Zhou R, Subramanyam G et al Human pallial MGE-type GABAergic interneuron cell therapy for chronic focal epilepsy. Cell Stem Cell 2023;30:1331–1350.e11. 37802038 10.1016/j.stem.2023.08.013 PMC10993865

[deag035-B12] Bondarenko V , Turco MY. Modelling the human maternal-fetal interface. Cell Stem Cell 2025;32:1321–1345. 10.1016/j.stem.2025.08.004 40912235

[deag035-B13] Boretto M , Cox B, Noben M, Hendriks N, Fassbender A, Roose H, Amant F, Timmerman D, Tomassetti C, Vanhie A et al Development of organoids from mouse and human endometrium showing endometrial epithelium physiology and long-term expandability. Development 2017;144:1775–1786. 28442471 10.1242/dev.148478

[deag035-B14] Burton GJ , Fowden AL, Thornburg KL. Placental origins of chronic disease. Physiol Rev 2016;96:1509–1565. 27604528 10.1152/physrev.00029.2015 PMC5504455

[deag035-B15] Cambridge center for Reproduction. *The SCBEM Code of Practice*. 2024.https://www.repro.cam.ac.uk/scbemcode (6 February 2026, date last accessed).

[deag035-B16] Cao J , Li W, Li J, Mazid MA, Li C, Jiang Y, Jia W, Wu L, Liao Z, Sun S et al Live birth of chimeric monkey with high contribution from embryonic stem cells. Cell 2023;186:4996–5014.e24. 37949056 10.1016/j.cell.2023.10.005

[deag035-B17] Carter AM. Animal models of human pregnancy and placentation: alternatives to the mouse. Reproduction 2020;160:R129–R143. 33112767 10.1530/REP-20-0354

[deag035-B18] Cave E. Advocating distinct regulatory paths for embryos and embryo-like structures. J Law Biosci 2025;12:lsaf008. 10.1093/jlb/lsaf008 40371268 PMC12074899

[deag035-B19] Chen C , Wu J, Wang X, Chang L, Wang K, Wu K, Guo M, Li H, Sun F, Jiang X et al Signaling reprogramming via Stat3 activation unravels high-fidelity human post-implantation embryo modeling. Cell Stem Cell 2025;32:1528–1544.e10. 40961945 10.1016/j.stem.2025.08.011

[deag035-B20] Clark AT , Brivanlou A, Fu J, Kato K, Mathews D, Niakan KK, Rivron N, Saitou M, Surani A, Tang F et al Human embryo research, stem cell-derived embryo models and *in vitro* gametogenesis: considerations leading to the revised ISSCR guidelines. Stem Cell Reports 2021;16:1416–1424. 34048690 10.1016/j.stemcr.2021.05.008 PMC8190666

[deag035-B21] Clark AT , Cook-Andersen H, Franklin S, Isasi R, Mathews DJH, Pasque V, Rugg-Gunn PJ, Tam PPL, Wang H, Zylicz JJ et al Stem cell-based embryo models: the 2021 ISSCR stem cell guidelines revisited. Stem Cell Reports 2025;20:102514. 10.1016/j.stemcr.2025.102514 40499509 PMC12181966

[deag035-B22] Coticchio G , Ahlstrom A, Arroyo G, Balaban B, Campbell A, De Los Santos MJ, Ebner T, Gardner DK, Kovacic B, Lundin K et al; The Working Group on the update of the ESHRE/ALPHA Istanbul Consensus. The Istanbul consensus update: a revised ESHRE/ALPHA consensus on oocyte and embryo static and dynamic morphological assessmentdagger, double dagger. Hum Reprod 2025;40:989–1035. 40288770 10.1093/humrep/deaf021 PMC12127515

[deag035-B23] Cyranoski D. How human embryonic stem cells sparked a revolution. Nature 2018;555:428–430. 29565377 10.1038/d41586-018-03268-4

[deag035-B24] de Graeff N , De Proost L. On the moral (in)equivalence of human embryos and stem cell-derived embryo models. J Med Ethics 2025;51:853–855. 40393700 10.1136/jme-2025-110866 PMC7617984

[deag035-B25] Deglincerti A , Etoc F, Guerra MC, Martyn I, Metzger J, Ruzo A, Simunovic M, Yoney A, Brivanlou AH, Siggia E et al Self-organization of human embryonic stem cells on micropatterns. Nat Protoc 2016;11:2223–2232. 27735934 10.1038/nprot.2016.131 PMC5821517

[deag035-B26] Dong C , Beltcheva M, Gontarz P, Zhang B, Popli P, Fischer LA, Khan SA, Park KM, Yoon EJ, Xing X et al Derivation of trophoblast stem cells from naive human pluripotent stem cells. Elife 2020;9. https://elifesciences.org/articles/52504 (6 February 2026, date last accessed). 10.7554/eLife.52504 PMC7062471 32048992

[deag035-B27] Fabbri M , Ginoza M, Assen L, Jongsma K, Isasi R. Modeling policy development: examining national governance of stem cell-based embryo models. Regen Med 2023;18:155–168. 36601984 10.2217/rme-2022-0136

[deag035-B28] Franklin S. Developmental landmarks and the Warnock report: a sociological account of biological translation. Comp Stud Soc Hist 2019;61:743–773.

[deag035-B29] Fu J , Warmflash A, Lutolf MP. Stem-cell-based embryo models for fundamental research and translation. Nat Mater 2021;20:132–144. 33199861 10.1038/s41563-020-00829-9 PMC7855549

[deag035-B30] Ghimire S , Mantziou V, Moris N, Arias AM. Human gastrulation: the embryo and its models. Dev Biol 2021;474:100–108. 33484705 10.1016/j.ydbio.2021.01.006

[deag035-B31] Gong Y , Bai B, Sun N, Ci B, Shao H, Zhang T, Yao H, Zhang Y, Niu Y, Liu L et al Ex utero monkey embryogenesis from blastocyst to early organogenesis. Cell 2023;186:2092–2110.e23. 37172563 10.1016/j.cell.2023.04.020

[deag035-B32] Health Council of the Netherlands. The 14-Day Rule in the Dutch Embryo Act (Publication No. 2023/16e). The Hague: Health Council of the Netherlands, 2023. https://www.healthcouncil.nl/documents/2023/10/31/the-14-day-rule-in-the-dutch-embryo-ac (6 February 2026, date last accessed).

[deag035-B33] Heemskerk I , Warmflash A. Pluripotent stem cells as a model for embryonic patterning: from signaling dynamics to spatial organization in a dish. Dev Dyn 2016;245:976–990. 27404482 10.1002/dvdy.24432

[deag035-B34] Hopwood N. Species choice and model use: reviving research on human development. J Hist Biol 2024;57:231–279. 39075321 10.1007/s10739-024-09775-7 PMC11341657

[deag035-B35] Human Fertilisation and Embryology Authority UK. *Fertility Treatment 2023: Trends and Figures*. 2025. https://www.hfea.gov.uk (6 February 2026, date last accessed).

[deag035-B36] Huntsman MC , Kurashima CK, Marikawa Y. Validation of a mouse 3D gastruloid-based embryotoxicity assay in reference to the ICH S5(R3) guideline chemical exposure list. Reprod Toxicol 2024;125:108558. 10.1016/j.reprotox.2024.108558 38367697 PMC11016378

[deag035-B37] Hyun I , Munsie M, Pera MF, Rivron NC, Rossant J. Toward guidelines for research on human embryo models formed from stem cells. Stem Cell Reports 2020;14:169–174. 31951813 10.1016/j.stemcr.2019.12.008 PMC7015820

[deag035-B38] Hyun I , Wilkerson A, Johnston J. Embryology policy: revisit the 14-day rule. Nature 2016;533:169–171. 27172031 10.1038/533169a

[deag035-B39] Rathenau Institute. *Holland’s Next Embryo Model: In Dialogue with Festival Visitors about Research with Embryo Models*. 2025. https://www.rathenau.nl/en/health/towards-responsible-medical-biotechnology/hollands-next-embryo-model (6 February 2026, date last accessed).

[deag035-B40] International Stem Cell Initiative. Assessment of established techniques to determine developmental and malignant potential of human pluripotent stem cells. Nat Commun 2018;9:1925. 29765017 10.1038/s41467-018-04011-3 PMC5954055

[deag035-B41] Ismaili MH. Is the integrated/non-integrated distinction for embryo models really obsolete? J Med Ethics 2025. 10.1136/jme-2025-111245 40903217

[deag035-B42] International Society for Stem Cell Research. *Guidelines for Stem Cell Research and Clinical Translation*. 2025. https://www.isscr.org/guidelines (6 February 2026, date last accessed).

[deag035-B43] Jarvis GE. Early embryo mortality in natural human reproduction: what the data say. F1000Res 2016;5:2765. 28580126 10.12688/f1000research.8937.1 PMC5443340

[deag035-B44] Jonlin EC , Fujita M, Isasi R, Kato K, Munsie M, Muto K, Niakan K, Saha K, Sugarman J, Turner L et al What does "appropriate scientific justification" mean for the review of human pluripotent stem cell, embryo, and related research? Stem Cell Reports 2025;20:102479. 10.1016/j.stemcr.2025.102479 40280137 PMC12143132

[deag035-B45] Jung E , Romero R, Yeo L, Gomez-Lopez N, Chaemsaithong P, Jaovisidha A, Gotsch F, Erez O. The etiology of preeclampsia. Am J Obstet Gynecol 2022;226:S844–S866. 35177222 10.1016/j.ajog.2021.11.1356 PMC8988238

[deag035-B46] Kagawa H , Javali A, Khoei HH, Sommer TM, Sestini G, Novatchkova M, Scholte Op Reimer Y, Castel G, Bruneau A, Maenhoudt N et al Human blastoids model blastocyst development and implantation. Nature 2022;601:600–605. 34856602 10.1038/s41586-021-04267-8 PMC8791832

[deag035-B47] Karvas RM , Zemke JE, Ali SS, Upton E, Sane E, Fischer LA, Dong C, Park KM, Wang F, Park K et al 3D-cultured blastoids model human embryogenesis from pre-implantation to early gastrulation stages. Cell Stem Cell 2023;30:1148–1165.e7. 37683602 10.1016/j.stem.2023.08.005

[deag035-B48] Kilens S , Meistermann D, Moreno D, Chariau C, Gaignerie A, Reignier A, Lelievre Y, Casanova M, Vallot C, Nedellec S et al; Milieu Intérieur Consortium. Parallel derivation of isogenic human primed and naive induced pluripotent stem cells. Nat Commun 2018;9:360. 10.1038/s41467-017-02107-w 29367672 PMC5783949

[deag035-B49] Kim J , Koo BK, Knoblich JA. Human organoids: model systems for human biology and medicine. Nat Rev Mol Cell Biol 2020;21:571–584. 32636524 10.1038/s41580-020-0259-3 PMC7339799

[deag035-B50] Kirkeby A , Main H, Carpenter M. Pluripotent stem-cell-derived therapies in clinical trial: a 2025 update. Cell Stem Cell 2025;32:329–331. 39864438 10.1016/j.stem.2025.01.003

[deag035-B51] Larsen EC , Christiansen OB, Kolte AM, Macklon N. New insights into mechanisms behind miscarriage. BMC Med 2013;11:154. 23803387 10.1186/1741-7015-11-154 PMC3699442

[deag035-B52] Li D , Wilcox AJ, Dunson DB. Benchmark pregnancy rates and the assessment of post-coital contraceptives: an update. Contraception 2015;91:344–349. 25592079 10.1016/j.contraception.2015.01.002 PMC4374046

[deag035-B53] Li H , Chang L, Wu J, Huang J, Guan W, Bates LE, Stuart HT, Guo M, Zhang P, Huang B et al *In vitro* generation of mouse morula-like cells. Dev Cell 2023;58:2510–2527.e7. 37875119 10.1016/j.devcel.2023.09.013

[deag035-B54] Li H , Guan W, Huang J, Shen P, Wu J, Luo H, Yang Y, Ning S, Chang L, Zhao H et al A complete model of mouse embryogenesis through organogenesis enabled by chemically induced embryo founder cells. Cell 2025;188:5912–5930.e20. 40780195 10.1016/j.cell.2025.07.018

[deag035-B55] Li J , Li J, Cao J, Shang S, Zhang L, Gao F, Fu J, Chen H, Cui G, Wu H et al Modelling late gastrulation in stem cell-derived monkey embryo models. Nature 2026;649:161–172. 41339550 10.1038/s41586-025-09831-0

[deag035-B56] Li J , Zhu Q, Cao J, Liu Y, Lu Y, Sun Y, Li Q, Huang Y, Shang S, Bian X et al Cynomolgus monkey embryo model captures gastrulation and early pregnancy. Cell Stem Cell 2023;30:362–377.e7. 37028403 10.1016/j.stem.2023.03.009

[deag035-B57] Li Q , Yuan Y, Zhao W, Li Y, Shi J, Xiu Y, Han M, Han Y, Zhang J, Cheng S et al A 3D *in vitro* model for studying human implantation and implantation failure. Cell 2026;189:70–86.e20. 41443192 10.1016/j.cell.2025.10.026

[deag035-B58] Li Y , He C, Yu H, Wu D, Liu L, Zhang X. Global, regional, and national epidemiology of congenital birth defects in children from 1990 to 2021: a cross-sectional study. BMC Pregnancy Childbirth 2025;25:484. 40275156 10.1186/s12884-025-07612-1 PMC12020332

[deag035-B59] Liu G , Li Y, Liu X, Wang W, Wen X, Che Y, Tu Q, Zhao B. Environmental nano-particulates induced pre-implantation embryonic toxicity in pluripotent stem cell-derived blastoids. Environ Pollut 2025;383:126809. https://www.sciencedirect.com/science/article/pii/S0269749125011820 (6 February 2026, date last accessed). 40645264 10.1016/j.envpol.2025.126809

[deag035-B60] Liu L , Oura S, Markham Z, Hamilton JN, Skory RM, Li L, Sakurai M, Wang L, Pinzon-Arteaga CA, Plachta N et al Modeling post-implantation stages of human development into early organogenesis with stem-cell-derived peri-gastruloids. Cell 2023;186:3776–3792.e16. 37478861 10.1016/j.cell.2023.07.018

[deag035-B61] Liu X , Nefzger CM, Rossello FJ, Chen J, Knaupp AS, Firas J, Ford E, Pflueger J, Paynter JM, Chy HS et al Comprehensive characterization of distinct states of human naive pluripotency generated by reprogramming. Nat Methods 2017;14:1055–1062. 28945704 10.1038/nmeth.4436

[deag035-B62] Liu X , Tan JP, Schröder J, Aberkane A, Ouyang JF, Mohenska M, Lim SM, Sun YBY, Chen J, Sun G et al Modelling human blastocysts by reprogramming fibroblasts into iBlastoids. Nature 2021;591:627–632. 33731926 10.1038/s41586-021-03372-y

[deag035-B63] Lovell-Badge R , Anthony E, Barker RA, Bubela T, Brivanlou AH, Carpenter M, Charo RA, Clark A, Clayton E, Cong Y et al ISSCR guidelines for stem cell research and clinical translation: the 2021 update. Stem Cell Reports 2021;16:1398–1408. 34048692 10.1016/j.stemcr.2021.05.012 PMC8190668

[deag035-B64] M’Hamdi HI , de Wert G. Reconsidering the 14-day rule in human embryo research: advice from the Dutch Health Council. Cell Stem Cell 2024;31:1560–1562. 39515299 10.1016/j.stem.2024.09.019

[deag035-B65] Mantziou V , Baillie-Benson P, Jaklin M, Kustermann S, Arias AM, Moris N. *In vitro* teratogenicity testing using a 3D, embryo-like gastruloid system. Reprod Toxicol 2021;105:72–90. 34425190 10.1016/j.reprotox.2021.08.003 PMC8522962

[deag035-B66] Marikawa Y. Toward better assessments of developmental toxicity using stem cell-based *in vitro* embryogenesis models. Birth Defects Res 2022;114:972–982. 35102709 10.1002/bdr2.1984 PMC9339025

[deag035-B67] Martinez Arias A , Marikawa Y, Moris N. Gastruloids: pluripotent stem cell models of mammalian gastrulation and embryo engineering. Dev Biol 2022;488:35–46. 35537519 10.1016/j.ydbio.2022.05.002 PMC9477185

[deag035-B68] Martinez Arias A , Rivron N, Moris N, Tam P, Alev C, Fu J, Hadjantonakis AK, Hanna JH, Minchiotti G, Pourquie O et al Criteria for the standardization of stem-cell-based embryo models. Nat Cell Biol 2024;26:1625–1628. 39223372 10.1038/s41556-024-01492-x

[deag035-B69] Matthews KR , Morali D. National human embryo and embryoid research policies: a survey of 22 top research-intensive countries. Regen Med 2020;15:1905–1917. 32799737 10.2217/rme-2019-0138

[deag035-B70] McCracken KW , Catá EM, Crawford CM, Sinagoga KL, Schumacher M, Rockich BE, Tsai Y-H, Mayhew CN, Spence JR, Zavros Y et al Modelling human development and disease in pluripotent stem-cell-derived gastric organoids. Nature 2014;516:400–404. 25363776 10.1038/nature13863 PMC4270898

[deag035-B71] Meng G , Gu J, Liew SY, Cao J, Wang Z, Ma C, Fu Z, Zhou H, Wang J, Wang S et al Reconstruction of endocrine subtype-complete human pluripotent stem cell-derived islets with capacity for hypoglycemia protection *in vivo*. Cell Stem Cell 2025;32:1438–1456.e7. 40782792 10.1016/j.stem.2025.07.006

[deag035-B72] Migliorini A , Nostro MC, Sneddon JB. Human pluripotent stem cell-derived insulin-producing cells: a regenerative medicine perspective. Cell Metab 2021;33:721–731. 33826915 10.1016/j.cmet.2021.03.021 PMC8117263

[deag035-B73] Miura Y , Li M-Y, Revah O, Yoon S-J, Narazaki G, Pașca SP. Engineering brain assembloids to interrogate human neural circuits. Nat Protoc 2022;17:15–35. 34992269 10.1038/s41596-021-00632-z

[deag035-B74] Mole MA , Elderkin S, Zorzan I, Penfold C, Horsley N, Pokhilko A, Polanek M, Palomar A, Sinha M, Wang Y et al Modeling human embryo implantation *in vitro*. Cell 2026;189:87–105.e28. 41443191 10.1016/j.cell.2025.10.027

[deag035-B75] Moore KL , Persaud TVN, Torchia MG. The Developing Human: Clinically Oriented Embryology, 11th edn. Philadelphia, PA: Elsevier, 2020.

[deag035-B76] Moris N , de Graeff N, Martinez Arias A, Pera M, Rivron N, Sermon K. Features of concern for ethical oversight in human embryology: a framework for decision-making by ethical committees overseeing embryo model research. Nat Cell Biol 2026, In press 10.1038/s41556-026-01881-4 PMC7619075 41951754

[deag035-B77] Ng ES , Sarila G, Li JY, Edirisinghe HS, Saxena R, Sun S, Bruveris FF, Labonne T, Sleebs N, Maytum A et al Long-term engrafting multilineage hematopoietic cells differentiated from human induced pluripotent stem cells. Nat Biotechnol 2025;43:1274–1287. 39223325 10.1038/s41587-024-02360-7 PMC12339382

[deag035-B78] National Health and Medical Research Council. *Regulation of Research Involving Human Embryos and Embryo-Like Structures in Australia*. 2023. https://www.nhmrc.gov.au/research-policy/embryo-research-licensing/commonwealth-and-state-legislation/determining-whether-embryo-model-regulated-erlc (6 February 2026, date last accessed).

[deag035-B79] Nuffield Council on Bioethics. *Human Embryo Culture*. 2017. https://www.nuffieldbioethics.org/wp-content/uploads/Human-Embryo-Culture-NCOB-paper-2017.pdf (6 February 2026, date last accessed).

[deag035-B80] Nuffield Council on Bioethics. *Human Stem Cell-Based Embryo Models: A Review of Ethical and Governance Questions*. https://cdn.nuffieldbioethics.org/wp-content/uploads/NCOB-SCBEM-Full-Report-Final.pdf (6 February 2026, date last accessed), 2024.

[deag035-B81] O’Rahilly R , Bossy J, Muller F. Introduction to the study of embryonic stages in man. Bull Assoc Anat (Nancy) 1981;65:141–236. 7030433

[deag035-B82] Oldak B , Wildschutz E, Bondarenko V, Comar MY, Zhao C, Aguilera-Castrejon A, Tarazi S, Viukov S, Pham TXA, Ashouokhi S et al Complete human day 14 post-implantation embryo models from naive ES cells. Nature 2023;622:562–573. 37673118 10.1038/s41586-023-06604-5 PMC10584686

[deag035-B83] Oura S , Li L, Wu J. An inducible model of human post-implantation development derived from primed and naive stem cells. Cell Stem Cell 2025;32:1509–1527.e9. 40885193 10.1016/j.stem.2025.08.005 PMC13325524

[deag035-B84] Pasca SP , Arlotta P, Bateup HS, Camp JG, Cappello S, Gage FH, Knoblich JA, Kriegstein AR, Lancaster MA, Ming GL et al A framework for neural organoids, assembloids and transplantation studies. Nature 2025;639:315–320. 39653126 10.1038/s41586-024-08487-6

[deag035-B85] Pastor WA , Chen D, Liu W, Kim R, Sahakyan A, Lukianchikov A, Plath K, Jacobsen SE, Clark AT. Naive human pluripotent cells feature a methylation landscape devoid of blastocyst or germline memory. Cell Stem Cell 2016;18:323–329. 26853856 10.1016/j.stem.2016.01.019 PMC4779431

[deag035-B86] Pera MF. Human embryo research and the 14-day rule. Development 2017;144:1923–1925. 28559237 10.1242/dev.151191

[deag035-B87] Pereira Daoud AM , Dondorp WJ, Bredenoord AL, de Wert G. The ethics of stem cell-based embryo-like structures: a focus group study on the perspectives of dutch professionals and lay citizens. J Bioeth Inq 2024;21:513–542. 38478325 10.1007/s11673-023-10325-9 PMC11652579

[deag035-B88] Popovic M , Dhaenens L, Taelman J, Dheedene A, Bialecka M, De Sutter P, Chuva de Sousa Lopes SM, Menten B, Heindryckx B. Extended *in vitro* culture of human embryos demonstrates the complex nature of diagnosing chromosomal mosaicism from a single trophectoderm biopsy. Hum Reprod 2019;34:758–769. 30838420 10.1093/humrep/dez012

[deag035-B89] Posfai E , Lanner F, Mulas C, Leitch HG. All models are wrong, but some are useful: establishing standards for stem cell-based embryo models. Stem Cell Reports 2021;16:1117–1141. 33979598 10.1016/j.stemcr.2021.03.019 PMC8185978

[deag035-B90] Pucelj M , Matusiak-Frącczak M. Global Perspectives on Reproductive Rights and Policies. Hershey, PA: IGI Global Scientific Publisher, 2024.

[deag035-B91] Ragusa D , Suen CW, Torregrosa Cortes G, Pastorino F, Johns A, Ciciro Y, Dijkhuis L, van den Brink S, Cilli M, Byrne C et al Dissecting infant leukemia developmental origins with a hemogenic gastruloid model. Elife 2025;14. https://elifesciences.org/articles/102324 (6 February 2026, date last accessed). 10.7554/eLife.102324 PMC12425479 40932369

[deag035-B92] Rivron N , Pera M, Rossant J, Martinez Arias A, Zernicka-Goetz M, Fu J, van den Brink S, Bredenoord A, Dondorp W, de Wert G et al Debate ethics of embryo models from stem cells. Nature 2018a;564:183–185. 30542177 10.1038/d41586-018-07663-9

[deag035-B93] Rivron NC , Frias-Aldeguer J, Vrij EJ, Boisset JC, Korving J, Vivie J, Truckenmuller RK, van Oudenaarden A, van Blitterswijk CA, Geijsen N. Blastocyst-like structures generated solely from stem cells. Nature 2018b;557:106–111. 29720634 10.1038/s41586-018-0051-0

[deag035-B94] Rivron NC , Martinez Arias A, Pera MF, Moris N, M’Hamdi HI. An ethical framework for human embryology with embryo models. Cell 2023a;186:3548–3557. 37595564 10.1016/j.cell.2023.07.028

[deag035-B95] Rivron NC , Martinez-Arias A, Sermon K, Mummery C, Scholer HR, Wells J, Nichols J, Hadjantonakis AK, Lancaster MA, Moris N et al Changing the public perception of human embryology. Nat Cell Biol 2023b;25:1717–1719. 37985870 10.1038/s41556-023-01289-4

[deag035-B96] Robinton DA , Daley GQ. The promise of induced pluripotent stem cells in research and therapy. Nature 2012;481:295–305. 22258608 10.1038/nature10761 PMC3652331

[deag035-B97] Rowe RG , Daley GQ. Induced pluripotent stem cells in disease modelling and drug discovery. Nat Rev Genet 2019;20:377–388. 30737492 10.1038/s41576-019-0100-z PMC6584039

[deag035-B98] Ruane PT , Garner T, Parsons L, Babbington PA, Wangsaputra I, Kimber SJ, Stevens A, Westwood M, Brison DR, Aplin JD. Trophectoderm differentiation to invasive syncytiotrophoblast is promoted by endometrial epithelial cells during human embryo implantation. Hum Reprod 2022;37:777–792. 35079788 10.1093/humrep/deac008 PMC9398450

[deag035-B99] Rugg-Gunn PJ , Moris N, Tam PPL. Technical challenges of studying early human development. Development 2023;150:dev201797. 10.1242/dev.201797 PMC10281548 37260362

[deag035-B100] Sadler TW. Establishing the embryonic axes: prime time for teratogenic insults. J Cardiovasc Dev Dis 2017;4:15. 10.3390/jcdd4030015 29367544 PMC5715709

[deag035-B101] Saitou M , Hayashi K. Mammalian *in vitro* gametogenesis. Science 2021;374:eaaz6830. 34591639 10.1126/science.aaz6830

[deag035-B102] Sasai Y. Next-generation regenerative medicine: organogenesis from stem cells in 3D culture. Cell Stem Cell 2013;12:520–530. 23642363 10.1016/j.stem.2013.04.009

[deag035-B103] Sasai Y , Eiraku M, Suga H. *In vitro* organogenesis in three dimensions: self-organising stem cells. Development 2012;139:4111–4121. 23093423 10.1242/dev.079590

[deag035-B104] Shahbazi MN , Jedrusik A, Vuoristo S, Recher G, Hupalowska A, Bolton V, Fogarty NNM, Campbell A, Devito L, Ilic D et al Self-organization of the human embryo in the absence of maternal tissues. Nat Cell Biol 2016;18:700–708. 27144686 10.1038/ncb3347 PMC5049689

[deag035-B105] Shahbazi MN , Siggia ED, Zernicka-Goetz M. Self-organization of stem cells into embryos: a window on early mammalian development. Science 2019;364:948–951. 31171690 10.1126/science.aax0164 PMC8300856

[deag035-B106] Shibata S , Endo S, Nagai LAE, H Kobayashi E, Oike A, Kobayashi N, Kitamura A, Hori T, Nashimoto Y, Nakato R et al Modeling embryo-endometrial interface recapitulating human embryo implantation. Sci Adv 2024;10:eadi4819. 38394208 10.1126/sciadv.adi4819 PMC10889356

[deag035-B107] Sidhaye J , Knoblich JA. Brain organoids: an ensemble of bioassays to investigate human neurodevelopment and disease. Cell Death Differ 2021;28:52–67. 32483384 10.1038/s41418-020-0566-4 PMC7853143

[deag035-B108] Siriwardena D , Boroviak TE. Evolutionary divergence of embryo implantation in primates. Philos Trans R Soc Lond B Biol Sci 2022;377:20210256. 10.1098/rstb.2021.0256 36252209 PMC9574630

[deag035-B109] Smith A. Propagating pluripotency—the conundrum of self-renewal. Bioessays 2024;46:e2400108. 10.1002/bies.202400108 39180242 PMC11589686

[deag035-B110] Smith GC. First-trimester determination of complications of late pregnancy. JAMA 2010;303:561–562. 20145237 10.1001/jama.2010.102

[deag035-B111] Song J , Zhao R, Zhang Y, Lu M, Liu P, Li T, Li C, Yu R, Chen X, Yang H et al 3D post-implantation co-culture of human embryo and endometrium. Cell Stem Cell 2026;33:58–72.e7. 41443195 10.1016/j.stem.2025.12.002

[deag035-B112] Statens medicinsk-etiska råd (Smer). *Embryon och embryomodeller–behovet av ett uppdaterat regelverk för forskning om det mänskliga livets tidiga utveckling*. smer.se. 2024. https://smer.se/wp-content/uploads/2024/04/smer-skrivelse-embryon-och-embryomodeller.pd (6 February 2026, date last accessed).

[deag035-B113] Steptoe PC , Edwards RG, Purdy JM. Human blastocysts grown in culture. Nature 1971;229:132–133. 4923103 10.1038/229132a0

[deag035-B114] Stern CD. Gastrulation: From Cells to Embryos. Cold Spring Harbor, NY: Cold Spring Harbour Laboratory Press, 2004.

[deag035-B115] Tabar V , Sarva H, Lozano AM, Fasano A, Kalia SK, Yu KKH, Brennan C, Ma Y, Peng S, Eidelberg D et al Phase I trial of hES cell-derived dopaminergic neurons for Parkinson’s disease. Nature 2025;641:978–983. 40240592 10.1038/s41586-025-08845-y PMC12095069

[deag035-B116] Tarazi S , Aguilera-Castrejon A, Joubran C, Ghanem N, Ashouokhi S, Roncato F, Wildschutz E, Haddad M, Oldak B, Gomez-Cesar E et al Post-gastrulation synthetic embryos generated ex utero from mouse naive ESCs. Cell 2022;185:3290–3306.e25. 35988542 10.1016/j.cell.2022.07.028 PMC9439721

[deag035-B117] The Lancet Global Health. Infertility—why the silence? Lancet Glob Health 2022;10:e773. 35561706 10.1016/S2214-109X(22)00215-7

[deag035-B118] Turco MY , Gardner L, Hughes J, Cindrova-Davies T, Gomez MJ, Farrell L, Hollinshead M, Marsh SGE, Brosens JJ, Critchley HO et al Long-term, hormone-responsive organoid cultures of human endometrium in a chemically defined medium. Nat Cell Biol 2017;19:568–577. 28394884 10.1038/ncb3516 PMC5410172

[deag035-B119] Turco MY , Gardner L, Kay RG, Hamilton RS, Prater M, Hollinshead MS, McWhinnie A, Esposito L, Fernando R, Skelton H et al Trophoblast organoids as a model for maternal-fetal interactions during human placentation. Nature 2018;564:263–267. 30487605 10.1038/s41586-018-0753-3 PMC7220805

[deag035-B120] Turner DA , Martinez Arias A. Three-dimensional stem cell models of mammalian gastrulation. Bioessays 2024;46:e2400123. 10.1002/bies.202400123 39194406 PMC11589689

[deag035-B121] van Mil H. Addressing the governance map: a public dialogue on the governance research involving stem cell-based embryo models. *A report*. University of Cambridge. 2024. https://static1.squarespace.com/static/56f16de77da24f3e5612733b/t/65389986b3acad2c463df88c/1698208160401/HVM+HDBI+public+dialogue+report+231023+FINAL.pdf (14 March 2026, date last accessed).

[deag035-B122] Vargesson N. Thalidomide-induced teratogenesis: history and mechanisms. Birth Defects Res C Embryo Today 2015;105:140–156. 26043938 10.1002/bdrc.21096 PMC4737249

[deag035-B123] Velasco S , Paulsen B, Arlotta P. 3D brain organoids: studying brain development and disease outside the embryo. Annu Rev Neurosci 2020;43:375–389. 32640930 10.1146/annurev-neuro-070918-050154

[deag035-B124] Wade JJ , MacLachlan V, Kovacs G. The success rate of IVF has significantly improved over the last decade. Aust N Z J Obstet Gynaecol 2015;55:473–476. 26174052 10.1111/ajo.12356

[deag035-B125] Wallace RL. The 14 day rule: scientific advances and the end of abortion rights. JSPG 2017;1.

[deag035-B126] Wang X , Wu Q. The divergent pluripotent states in mouse and human cells. Genes (Basel) 2022;13:773. 10.3390/genes13081459 PMC9408542 36011370

[deag035-B127] Warnock M. *Report of the Committee of Inquiry into Human Fertilisation and Embryology*, (Cmnd. 9314). Her Majesty's Stationery Office, 1984.

[deag035-B128] Wilcox AJ , Dunson DB, Weinberg CR, Trussell J, Baird DD. Likelihood of conception with a single act of intercourse: providing benchmark rates for assessment of post-coital contraceptives. Contraception 2001;63:211–215. 11376648 10.1016/s0010-7824(01)00191-3

[deag035-B129] Wu W , He J, Shao X. Incidence and mortality trend of congenital heart disease at the global, regional, and national level, 1990-2017. Medicine (Baltimore) 2020;99:e20593. 32502030 10.1097/MD.0000000000020593 PMC7306355

[deag035-B130] Xu J , Li Q, Deng L, Xiong J, Cheng Z, Ye C. Global, regional, and national epidemiology of congenital heart disease in children from 1990 to 2021. Front Cardiovasc Med 2025;12:1522644. 40454242 10.3389/fcvm.2025.1522644 PMC12122482

[deag035-B131] Yamanaka Y , Hamidi S, Yoshioka-Kobayashi K, Munira S, Sunadome K, Zhang Y, Kurokawa Y, Ericsson R, Mieda A, Thompson JL et al Reconstituting human somitogenesis *in vitro*. Nature 2023;614:509–520. 36543322 10.1038/s41586-022-05649-2

[deag035-B132] Yanagida A , Spindlow D, Nichols J, Dattani A, Smith A, Guo G. Naive stem cell blastocyst model captures human embryo lineage segregation. Cell Stem Cell 2021;28:1016–1022.e4. 33957081 10.1016/j.stem.2021.04.031 PMC8189436

[deag035-B133] Yilmaz A , Gurhan G, Comar MY, Viukov S, Serfaty I, Gayretli M, Golenchenko S, Lokshtanov D, Ashouokhi S, Polanco A et al Transgene-free generation of mouse post-gastrulation whole embryo models solely from naive ESCs and iPSCs. Cell Stem Cell 2025;32:1545–1562.e12. 40780191 10.1016/j.stem.2025.07.005

[deag035-B134] Yu L , Wei Y, Duan J, Schmitz DA, Sakurai M, Wang L, Wang K, Zhao S, Hon G, Wu J. Blastocyst-like structures generated from human pluripotent stem cells. Nature 2021;591:620–626. 33731924 10.1038/s41586-021-03356-y

[deag035-B135] Zhai J , Xu Y, Wan H, Yan R, Guo J, Skory R, Yan L, Wu X, Sun F, Chen G et al Neurulation of the cynomolgus monkey embryo achieved from 3D blastocyst culture. Cell 2023;186:2078–2091.e18. 37172562 10.1016/j.cell.2023.04.019

[deag035-B136] Zhang X , An S, Liu S, Qiu J, Zhang W, Zhou Q, Hou X, Yang Y. Comparative assessment of embryotoxicity of 2,4,6-triiodophenol to mouse blastoid and pre-implantation embryo models. Ecotoxicol Environ Saf 2023;252:114608. 10.1016/j.ecoenv.2023.114608 36738612

[deag035-B137] Zhao C , Plaza Reyes A, Schell JP, Weltner J, Ortega NM, Zheng Y, Bjorklund AK, Baque-Vidal L, Sokka J, Trokovic R et al A comprehensive human embryo reference tool using single-cell RNA-sequencing data. Nat Methods 2025;22:193–206. 39543283 10.1038/s41592-024-02493-2 PMC11725501

